# Identification of Differentially-Expressed Genes Associated with Pistil Abortion in Japanese Apricot by Genome-Wide Transcriptional Analysis

**DOI:** 10.1371/journal.pone.0047810

**Published:** 2012-10-16

**Authors:** Ting Shi, Zhihong Gao, Liangju Wang, Zhen Zhang, Weibing Zhuang, Hailong Sun, Wenjun Zhong

**Affiliations:** College of Horticulture, Nanjing Agricultural University, Nanjing, People's Republic China; Kyushu Institute of Technology, Japan

## Abstract

The phenomenon of pistil abortion widely occurs in Japanese apricot, and imperfect flowers with pistil abortion seriously decrease the yield in production. Although transcriptome analyses have been extensively studied in the past, a systematic study of differential gene expression has not been performed in Japanese apricot. To investigate genes related to the pistil development of Japanese apricot, high-throughput sequencing technology (Illumina) was employed to survey gene expression profiles from perfect and imperfect Japanese apricot flower buds. 3,476,249 and 3,580,677 tags were sequenced from two libraries constructed from perfect and imperfect flower buds of Japanese apricot, respectively. There were 689 significant differentially-expressed genes between the two libraries. GO annotation revealed that highly ranked genes were those implicated in small molecule metabolism, cellular component organisation or biogenesis at the cellular level and fatty acid metabolism. According to the results, we assumed that late embryogenesis abundant protein (LEA), Dicer-like 3 (DCL3) Xyloglucan endotransglucosylase/hydrolase 2 (XTH2), Pectin lyase-like superfamily protein (PPME1), Lipid transfer protein 3 (LTP3), Fatty acid biosynthesis 1 (FAB1) and Fatty acid desaturase 5 (FAD5) might have relationships with the pistil abortion in Japanese apricot. The expression patterns of 36 differentially expressed genes were confirmed by real-time (RT)-PCR. This is the first report of the Illumina RNA-seq technique being used for the analysis of differentially-expressed gene profiles related to pistil abortion that both computationally and experimentally provides valuable information for the further functional characterisation of genes associated with pistil development in woody plants.

## Introduction

Japanese apricot (*Prunus mume* Sieb. et Zucc) belongs to the *Rosaceae* family of fruits and is an important economic fruit crop in China and Japan [1]. Owing to its high nutritional value, the fruit has not only been used in the preparation of preserved fruit and wine, but can also be used as a diet ingredient [1], [2]. However, the phenomenon of imperfect flowers widely occurs and has seriously affected the production yield [3]. The imperfect flowers are characterised by either pistils below the stamens, withered pistils or an absence of pistils, and such flowers fail to bear fruit [4]. Comparative proteomic analysis has been performed for perfect and imperfect flowers and the different proteins have been analysed in both perfect and imperfect flowers for the different stages of young bud, mature bud and blossom flower; moreover, glucose metabolism, starch metabolism and photosynthesis related to pistil abortion were found [5]. More recently, real-time quantitative reverse transcription polymerase chain reactions and *in situ* hybridisation have shown that *PmAG* mRNA was highly expressed in the sepals, carpel and stamens, and a weak signal was detected in the seed and nutlet. No expression was detected in the leaves or petals, but no significant differential expression was found between perfect and imperfect flowers [4]. In addition, morphological research indicated that during the first and second ten days of December, flower buds of the ‘Daqiandi’ cultivar did not continue to elongate, instead the pistil differentiation stagnated and gradually disintegrated, which was the key stage of pistil abortion of the ‘Daqiandi’ cultivar. The factors leading to the selective abortion of pistils may relate to catabolism of macromolecule nutrients in the flower bud [6]. However, the molecular mechanism involved in pistil abortion remains unknown for Japanese apricot.

In most floral plants, floral organs play a significant role in plant sexual reproduction, but only a few of the flowers and ovules that are initiated actually give rise to mature seeds and fruits [7]. Several different mechanisms have been proposed to explain the phenomenon of female sterility, including pistil abortion. Female sterility is thought to be triggered by environmental and nutritional conditions [8], [9], low sink strength [10], [11], the influence of pathogens [12], the occurrence of sporophytic or gametophytic mutations [13], ABCDE model and other related genes [14]–[16] and phytohermone [17]–[20]. Morphological studies have shown that pistil development of staminate flowers in the olive is interrupted after differentiation of the megaspore mother cell. At that stage, no starch was observed in the pistils of the staminate flowers; the plastids had few thylakoid membranes and grana and the staminate flowers appeared very similar to proplastids [10]. In *Arabidopsis*, heat-stress reduced the total number of ovules and increased ovule abortion [21]. Early ovule degeneration was also caused by high temperatures in sweet cherry, and ovule development was regulated by gibberellin (GA) in sweet cherry flowers [9]. GA suppressed the development of the embryo sac and shortened its longevity in grapes [20].

Next-generation high-throughput sequencing technologies provide a powerful strategy to identify genome-wide expression. Genome-wide expression analyses are essential tools for elucidating molecular function. Recent studies have highlighted the significance of high-throughput expression data, particularly with the integration of large, diverse data sets, in constructing biochemical and regulatory networks *in silico* [22]–[25]. Digital gene expression tag profiling (DGE) is a revolutionary approach for expression analysis [26], [27]. Driven by Solexa/Illumina technology, DGE creates genome-wide expression profiles by sequencing. This method combines the sequencing serial analysis of gene expression (SAGE) principle with the sequencing technology for generating a digital output proportional to the number of transcripts per mRNA [28]. The benefit of not requiring pre-synthesised oligonucleotide probes (as in microarrays) allows the direct enumeration of transcripts, which is highly replicable, accurate and comparable across experiments [29], [30]. In addition, the quantification of weakly expressed genes can be performed, which cannot be assessed using microarrays [31]. DGE also offers researchers a global orthogonal hybridisation array validation method, with almost unlimited dynamic range, providing a tuneable depth of coverage for rare transcript discovery and quantification. For example, to explore the molecular mechanism of plant developmental-stage transition and cell-fate determination, DGE analysis was undertaken of sequential developmental time-points and individual tissue types in the model moss *Physcomitrella patens* [24], which was chosen because of the short life-cycle and relative structural simplicity of this plant. To validate gene expression in the models for trichome development in soybean, DGE analysis and RNA-Seq were used to compare the transcriptional profiles in wild-type and glabrous soybean lines [32]. Early developing cotton fibre was analysed by deep sequencing, and differential expressions of genes in a fuzzless/lintless mutant were revealed [33]. DGE signatures were also used to study the maize *RA3* gene [22] and the development of brace root [34]. In addition, Solexa/Illumina technology was used to analyse gene expression during female flower development in cucumber [35]. Overall, the DGE approach has provided more valuable tools for qualitative and quantitative gene expression analysis than the previous microarray-based assays [26].

Here, we used the DGE method to perform a deep transcriptome analysis of pistil abortion in Japanese apricot. Although transcriptome analyses have been extensively studied in the past years, for example, 454-pyrosequencing of the transcriptome was used in the research of dormant stages in Japanese apricot [36], a systematic study of differential gene expression has not been carried out on Japanese apricot. To investigate the differentially-expressed genes related to the pistil development of Japanese apricot, high-throughput sequencing technology (Solexa) was employed to survey the gene expression profiles from perfect and imperfect Japanese apricot flower buds.

## Results

### Statistics of tag sequencing

**Figure 1:**
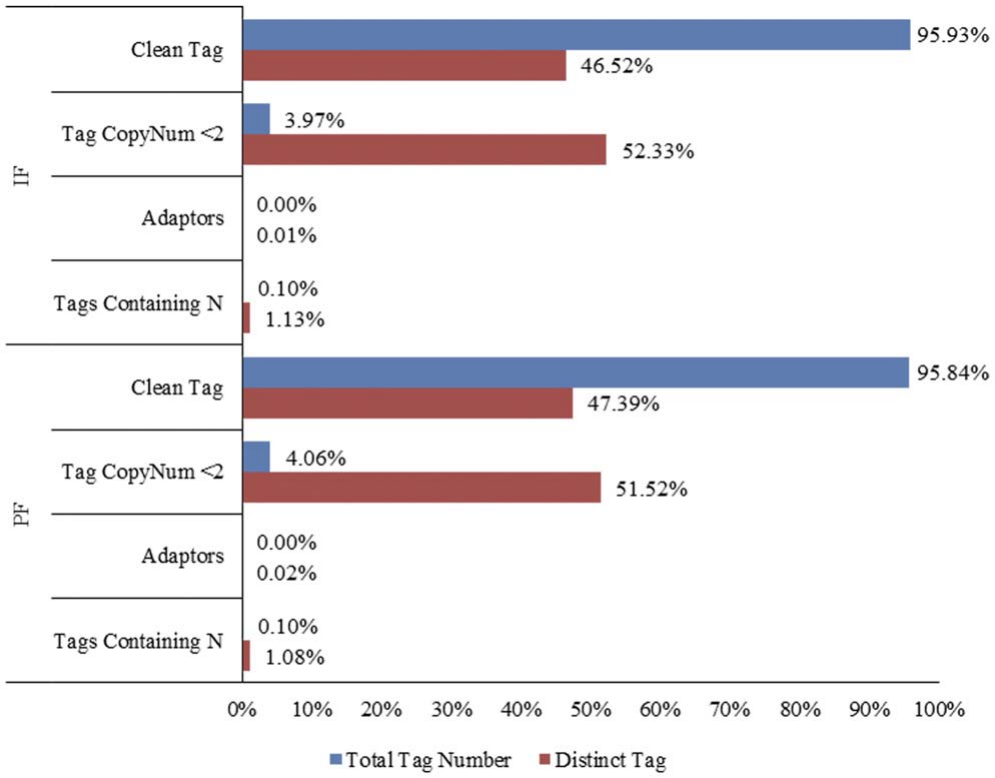
Distribution of tag expression. Left is PF and right is IF. Total clean tags represent the sum of all clean tag numbers; distinct clean tags represent all types of clean tags.

To identify differentially-expressed genes involved in the pistil development of flowers in Japanese apricot, we used Illumina sequencing on DGE from the perfect (PF) and imperfect (IF) flower buds. A total of 3,476,249 and 3,580,677 tags were obtained from the PF and IF flower bud libraries, respectively (Table 1). To increase the robustness of the approach, single-copy tags in the two libraries (141,270 in the PF and 142,287 in the IF library) were excluded from further analysis (Figure 1). After discarding the low quality tags (tags containing ‘N’, adaptor sequences and copy number <2), 3,331,468 and 3,434,800 tags (clean tags) remained in the PF and IF libraries, from which 129,933 (PF) and 126,485 (IF) distinct tags were obtained (Figure 1). There were 3,448 more distinct tags in the PF than in the IF library, possibly representing genes related to pistil development. The percentage of distinct tags rapidly declined as copy number increased, indicating that only a small portion of the transcripts were expressed at a high level under the conditions tested.

**Table 1:** Statistics of DGE sequencing.

Summary		PF	IF
Raw Data	Total	3,476,249	3,580,677
Raw Data	Distinct Tag	274,206	271,884
Clean Tag	Total number	3,331,468	3,434,800
Clean Tag	Distinct Tag number	129,933	126,485
All Tag Mapping to Gene	Total number	1783091	1703889
All Tag Mapping to Gene	Total % of clean tag	53.52%	49.61%
All Tag Mapping to Gene	Distinct Tag number	64599	58309
All Tag Mapping to Gene	Distinct Tag % of clean tag	49.72%	46.10%
Unambiguous Tag Mapping to Gene	Total number	1564689	1496735
Unambiguous Tag Mapping to Gene	Total % of clean tag	46.97%	43.58%
Unambiguous Tag Mapping to Gene	Distinct Tag number	57871	52144
Unambiguous Tag Mapping to Gene	Distinct Tag % of clean tag	44.54%	41.23%
All Tag-mapped Genes	number	17056	16386
All Tag-mapped Genes	% of ref genes	59.45%	57.12%
Unambiguous Tag-mapped Genes	number	14106	13486
Unambiguous Tag-mapped Genes	% of ref genes	49.17%	47.01%
Mapping to Genome	Total number	776068	849241
Mapping to Genome	Total % of clean tag	23.30%	24.72%
Mapping to Genome	Distinct Tag number	34698	34806
Mapping to Genome	Distinct Tag % of clean tag	26.70%	27.52%
Unknown Tag	Total number	772309	881670
Unknown Tag	Total % of clean tag	23.18%	25.67%
Unknown Tag	Distinct Tag number	30636	33370
Unknown Tag	Distinct Tag % of clean tag	23.58%	26.38%

**Figure 2:**
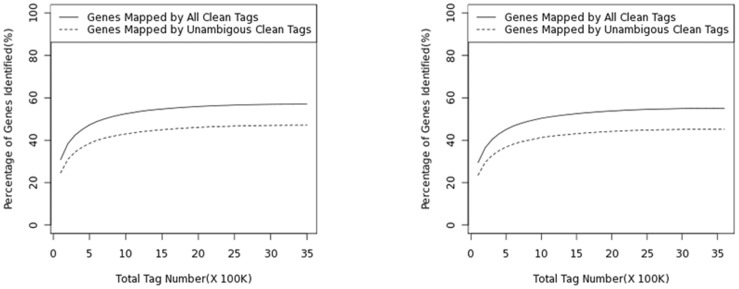
Saturation evaluation of differential expression. The left figure is PF and the right figure is IF.

The saturation of the library was determined by the identification of unique tags. Sequencing was considered to have reached saturation when no new unique tags were detected. The results shown in Figure 2 indicate that the PF and IF libraries were sequenced to saturation, producing a full representation of the transcripts in the conditions tested. In both libraries, fewer unique tags were identified as the number of sequencing tags increased, reaching a plateau shortly after 1 M tags were sequenced. No new unique tags were identified as the total tag number approached 3 M in the PF library and 2.5 M in the IF library.

**Figure 3:**
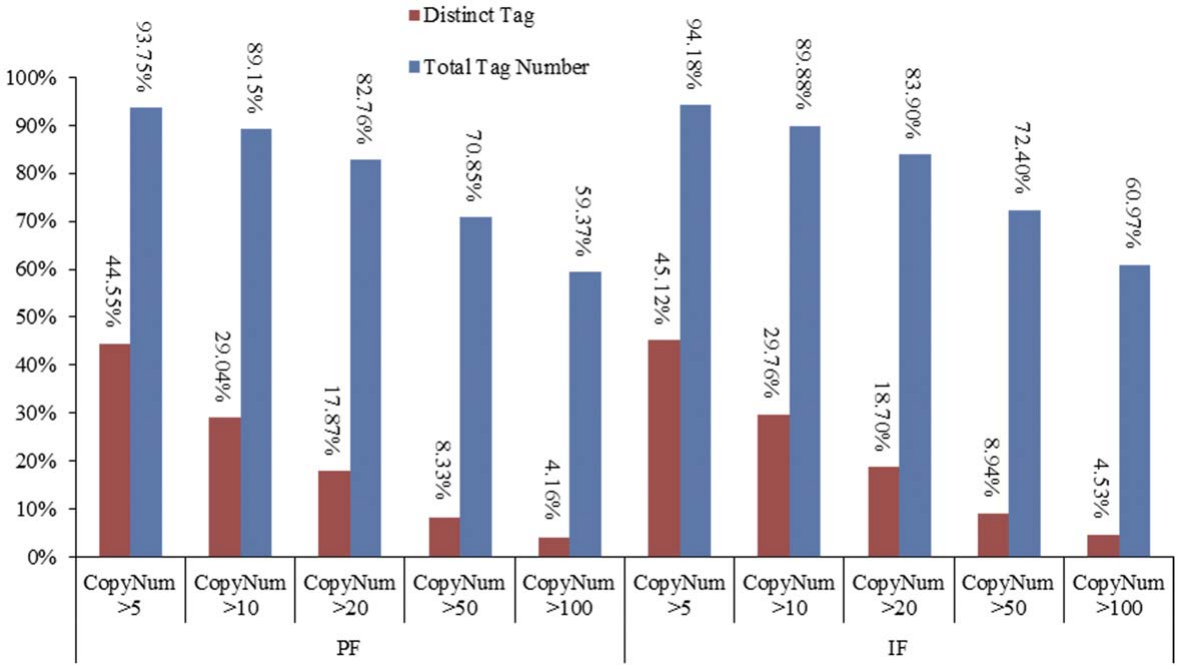
Distribution of clean tag copy number. Left is PF and right is IF. Total clean tags represent the sum of all clean tag numbers; distinct clean tags represent all types of clean tags.

The distribution of the clean tag copy number is shown in figure 3. The copy number of clean tags between 2 and 5 was 208,111, between 6 and 10 was 153,194, between 11 and 20 was 213,051, between 21 and 50 was 396,739, between 51 and 100 was 382,603, and >100 was 1,977,770 in the PF library. In contrast, the copy number of distinct clean tags was mostly distributed between 2 and 5, with 55.45% of the total clean tags in the PF library (Figure 3). In the IF library, the copy number of clean tags between 2 and 5 was 199,910, between 6 and 10 was 147,600, between 11 and 20 was 205,594, between 21 and 50 was 395,033, between 51 and 100 was 392,318, and >100 was 2,094,345. Similar to the PF library, the copy number of distinct clean tags was mostly distributed between 2 and 5, with 54.88% of the total clean tags in the IF library (Figure 3).

### Annotation analysis of the unique tags

**Figure 4:**
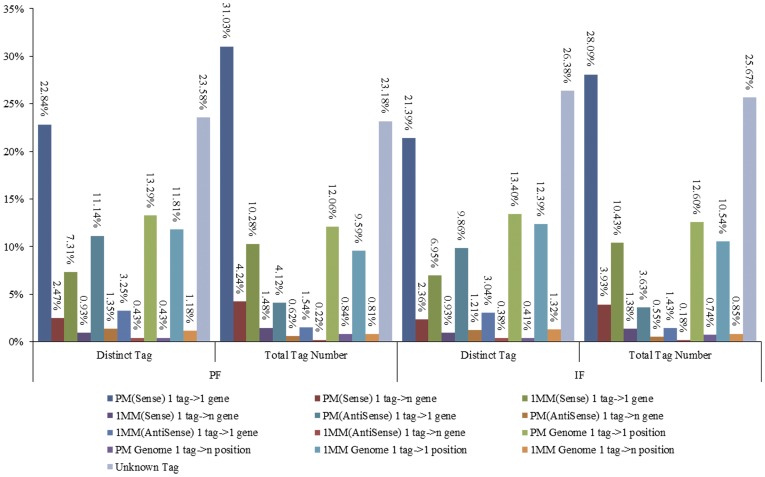
Alignment results of the clean tags. The figure exhibits the alignment statistics of the tag copy number and the tag type. Note: 1PM (sense or antisense): perfect match to gene (sense or antisense); 1 tag->1 gene: match to one gene; 1 tag->n gene: match to more than one gene; 1 MM (sense or antisense): match to gene (sense or antisense) with 1bp mismatch; PM Genome 1 tag->1 position: perfect match to genome sequence with one best hit; PM Genome 1 tag->n position: perfect match to genome sequence with multiple best hits; 1 MM Genome: match to genome sequence with 1bp mismatch; Unknown Tag: no match to gene (sense and anti-sense) and genome sequence.

The distinct tags were compared against the genome and gene sequences of peach using BLASTn. Tags with a complete match or one base-pair mismatch were considered further. The results in Table 1 show that a proportion of tags (26.70% in the PF library and 27.52% in the IF library) matched to the peach genome, but 64,599 (49.72% of unique tags) and 58,309 (46.10% of unique tags) in the PF and IF libraries matched to 17,056 (59.45%) and 16,386 (57.12%) peach genes. Further analysis revealed that 57,871 unique tags (44.54%) in the PF library and 52,144 (41.23%) in the IF library matched to only one gene sequence in the peach genome (Table 1 and Figure 4), including perfect matching to genes and with a 1bp mismatch. These data indicate that approximately 50% of the transcripts predicted in Japanese apricot are expressed in the perfect or imperfect flowers, with more transcripts present in the perfect sample. Figure 4 shows that sense regulation, with clean sequencing tags perfectly mapped to the sense gene being 22.84% (antisense was 11.14%) and 21.39% (antisense was 9.86%) in PF and IF, respectively, was the main regulated model.

### Gene ontology functional enrichment analysis of differentially-expressed genes (DEGs)

**Figure 5:**
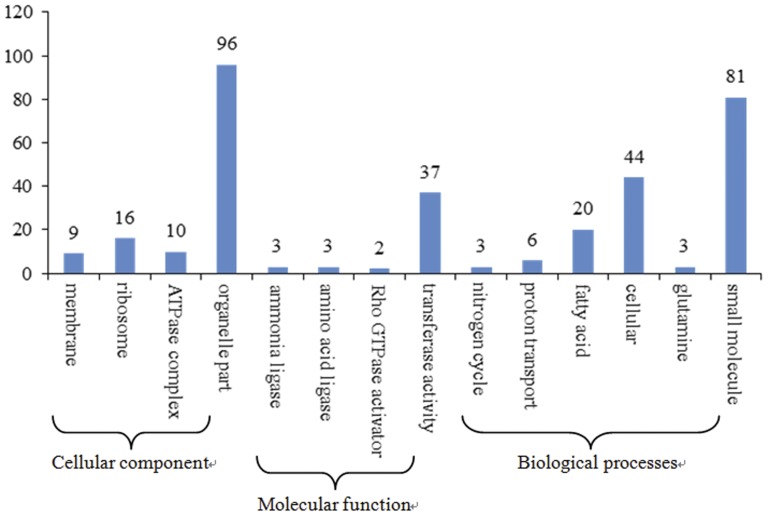
Histogram showing the Gene Ontology functional enrichment of DEGs.

Using the P-value ≤0.05 as the threshold value, 333 differentially expressed genes that could be categorised into 14 functional groups were found (Figure 5), which included four cellular components, four molecular functions and six biological processes. The four component categories were as follows: anchored to membrane (9), ribosome (16), ATPase complex (10) and organelle part (96). Based on molecular function, the genes were finally classified into four categories: ammonia ligase activity (3), acid-ammonia (or amide) ligase activity (3), Rho GTPase activator activity (2) and transferase activity (37). Additionally, six biological processes were identified: nitrogen cycle metabolism (3), proton transport (6), fatty acid metabolism (20), cellular component organisation or biogenesis at the cellular level (44), glutamine metabolism (3) and small molecule metabolism (81). The genes involved in small molecule metabolism [GO: 0044281] were the most significantly enriched in comparison to the other five biological processes. Forty-four differentially expressed genes were involved in the cellular component organisation or biogenesis at the cellular level [GO: 0071841], which is carried out at the cellular level and results in the biosynthesis of constituent macromolecules, assembly, arrangement of constituent parts, or disassembly of a cellular component. Among the significantly enriched transcripts were 20 DEGs associated with the regulation of fatty acid metabolism [GO: 0006631], which includes the chemical reactions and pathways involving fatty acids and aliphatic monocarboxylic acids liberated from naturally occurring fats and oils by hydrolysis.

### Pathway enrichment analysis for DEGs

**Figure 6:**
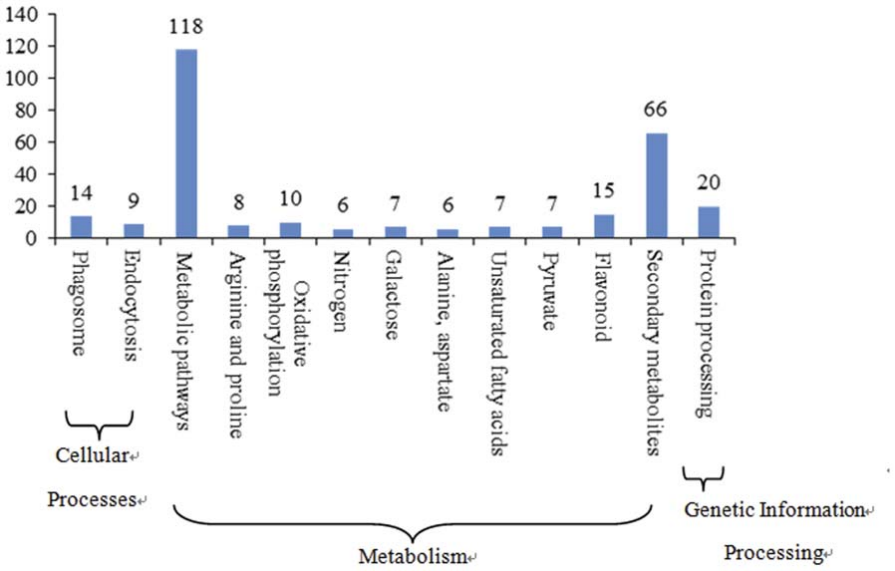
Histogram illustrating pathway enrichment analyses.

A Q-value of ≤0.05 defined those genes that were significantly differentially expressed (enriched). 227 differentially expressed genes associated with 12 metabolic and signal transduction pathways were found (Figure 6). The pathways with the most unique genes were metabolic pathways (250 genes), cellular process (66 genes) and genetic information processing (20 genes). We believe that these pathways are significant in the pistil abortion of Japanese apricot, in particular metabolic pathways and cellular processes. In our study, metabolic pathways (ko01100) are large complexes comprising several metabolic patterns, including: the biosynthesis of secondary metabolites (ko01110, 66 genes); flavonoid biosynthesis (ko00941, 15); oxidative phosphorylation (ko00190, 10 genes); arginine and proline metabolism (ko00330, eight genes); galactose metabolism (ko00052, seven genes); the biosynthesis of unsaturated fatty acids (ko01040, seven genes); pyruvate metabolism (ko00620, seven genes); nitrogen metabolism (ko00910, six genes) and alanine, aspartate and glutamate metabolism (ko00330, six genes). Cellular processes included phagosome (ko04145, 14 genes) and endocytosis (ko04144, nine genes). Genetic information processing only included protein processing in the endoplasmic reticulum (ko04141, 20 genes).

### Comparison of gene expression between the two libraries

**Figure 7:**
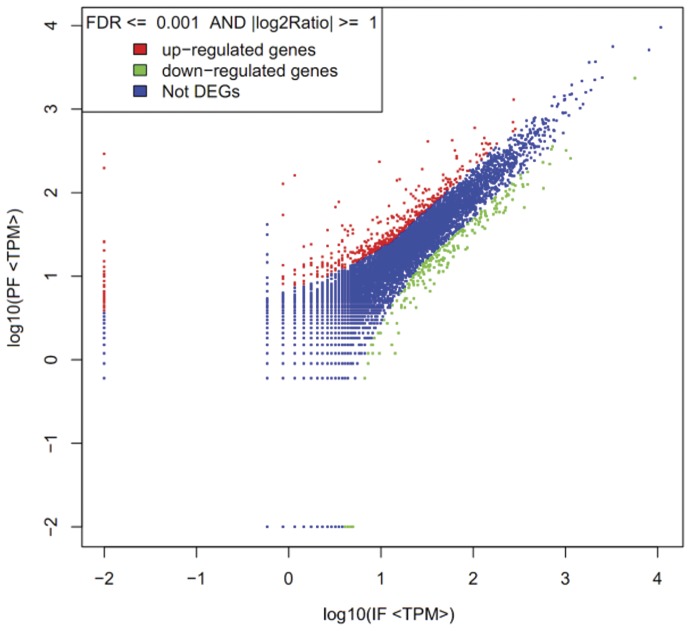
Comparison of gene expression levels between the two libraries. For comparing gene expression levels between the two libraries, each library was normalised to 1 million tags. Red dots represent transcripts more prevalent in the infected leaf library, green dots show those present at a lower frequency in the infected tissue and blue dots indicate transcripts that did not change significantly. The parameters FDR <0.001 and log2 ratio ≥ 1 were used as the threshold to judge the significance of gene expression difference.

**Figure 8:**
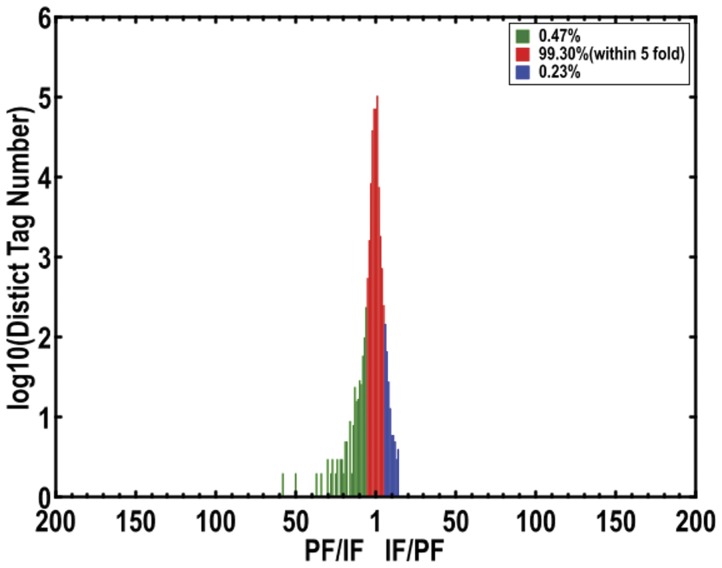
Differentially-expressed tags in the IF tissue library. The x-axis represents fold-change of differentially expressed unique tags in the imperfect library. The y-axis represents the number of unique tags (log10). Differentially accumulating unique tags with a five-fold difference between libraries are shown in the red region (99.30%). The blue (0.23%) and green (0.47%) regions represent unique tags that are up- and down-regulated more than five-fold in the IF library, respectively.

Differences in the tag frequencies that appeared in the PF and IF libraries were used to estimate gene expression levels in response to pistil abortion. The transcripts detected with at least a two-fold difference between the two libraries are shown in Figure 7 (FDR <0.001). The red dots (468) and green dots (221) represent transcripts with a higher or lower abundance of more than two-fold than in the PF library, respectively. The blue dots represent transcripts that differed less than two-fold between the two libraries, which were arbitrarily designated as “no difference in expression”. The differentially-expressed genes with five-fold or greater differences in accumulation are shown in Figure 8 and Table 2. About 0.23% total unique tags increased by at least five-fold, and about 0.47% total unique tags were decreased by at least five-fold in the IF library, while the expression level of 99.3% unique tags was within the five-fold difference between the two samples.

**Table 2:** List of differentially expressed genes changed by five-fold or more in the IF library.

Gene	log2 Ratio(IF/PF)	Description
Up-regulated genes		
Defence		
ppa004141m	8.86	Calcium-dependent protein kinase 13 (CPK13)
ppa018301m	8.86	CPR30 F-box and associated interaction domains-containing protein
Metabolism		
ppa000615m	8.86	EMB3011 RNA helicase family protein
ppa010131m	8.67	Peptidyl-tRNA hydrolase family protein
ppa006743m	8.67	AGD2 Pyridoxal phosphate (PLP)-dependent transferases superfamily protein
ppa006182m	8.86	Protein kinase superfamily protein
ppa021659m	8.95	Dicer-like 3 (DCL3)
ppa005799m	8.77	Pre-mRNA-splicing factor CWC26
Signal transduction		
ppa012743m	8.77	SLY2 F-box family protein
ppa003105m	8.67	Leucine-rich repeat protein kinase family protein
Transport		
ppa013294m	8.95	Vacuolar ATPase subunit F family protein
Down-regulated genes		
Defence		
ppa002249m	−8.61	Early-responsive to dehydration stress protein (ERD4)
ppa016718m	−8.99	ENODL5 early nodulin-like protein 5
ppa002203m	−9.37	FRO2 ferric reduction oxidase 2
ppa006485m	−9.61	Mitogen-activated protein kinase kinase kinase 15 (MAPKKK15)
ppa021261m	−10.04	Late embryogenesis abundant protein (LEA) family protein
Development		
ppa001970m	−8.71	Glutamine-rich protein 23 (GRP23)
ppa016920m	−8.99	BTB/POZ domain-containing protein
Metabolism		
ppa023612m	−5.96	Lipid transfer protein 3 (LTP3)
ppa011478m	−7.11	Plant invertase/pectin methylesterase inhibitor superfamily protein
ppa023515m	−7.18	Cysteine proteinases superfamily protein
ppa009726m	−8.61	ABI-1-like 1 (ABIL1)
ppa025960m	−8.61	BEN1 NAD(P)-binding Rossmann-fold superfamily protein
ppa015204m	−8.61	SYD P-loop containing nucleoside triphosphate hydrolases superfamily protein
ppa002676m	−8.61	Pentatricopeptide repeat (PPR) superfamily protein
ppa005209m	−8.81	XF1 FAD/NAD(P)-binding oxidoreductase family protein
ppa026851m	−8.81	Subtilisin-like serine endopeptidase family protein
ppa007640m	−8.91	IRX9 Nucleotide-diphospho-sugar transferases superfamily protein
ppa003553m	−8.91	P-loop containing nucleoside triphosphate hydrolases superfamily protein
ppa022113m	−8.91	3-ketoacyl-CoA synthase 7 (KCS7)
ppb019226m	−9.08	Plant invertase/pectin methylesterase inhibitor superfamily protein
ppa007503m	−9.15	PLC-like phosphodiesterases superfamily protein
ppa017270m	−9.3	Alpha/beta-Hydrolases superfamily protein
ppa005976m	−9.55	Pectin lyase-like superfamily protein (PPME1)
ppa018639m	−9.61	Cytochrome P450, family 735, subfamily A, polypeptide 1 (CYP735A1)
ppa013439m	−9.76	RING/U-box superfamily protein
ppa004479m	−9.95	Fatty acid biosynthesis 1 (FAB1)
ppa020149m	−10.16	Alpha dioxygenase
ppa005749m	−10.55	Purple acid phosphatase 22 (PAP22)
ppa004364m	−10.55	Non-specific phospholipase C3 (NPC3)
ppa019741m	−10.97	Xyloglucan endotransglucosylase/hydrolase 2 (XTH2)
ppa025833m	−11.32	Alpha/beta-Hydrolases superfamily protein
ppa020405m	−11.33	GDSL-like Lipase/Acylhydrolase superfamily protein
ppa027208m	−14.26	Fatty acid desaturase 5 (FAD5)
ppa016543m	−14.82	Fatty acid desaturase 5 (FAD5)
Signal transduction		
ppa015093m	−8.91	Cyclin D6
ppa016219m	−8.91	Stigma-specific Stig1 family protein
ppa012463m	−9.61	Pollen Ole e 1 allergen and extensin family protein
Transcription		
ppa011751m	−8.61	Myb domain protein 24 (MYB24)
ppa008450m	−9.23	Myb domain protein 73 (MYB73)
Transport		
ppa004487m	−8.61	MATE efflux family protein
ppa010364m	−8.61	TIP1;3 tonoplast intrinsic protein 1;3
ppa000945m	−8.81	HA9 H(+)−ATPase 9
ppa006913m	−9.61	AAC2 ADP/ATP carrier 2
Various functions		
ppa014641m	−8.71	Plant protein of unknown function (DUF868)
ppa020792m	−8.71	ARM repeat superfamily protein
ppa022037m	−8.81	Uncharacterized protein family (UPF0016)
ppa014616m	−8.81	Protein of unknown function (DUF1278)
ppa021534m	−10.16	Protein of unknown function (DUF679)
ppa008861m	−10.33	Family of unknown function (DUF716)

Of DEGs with differences greater than five-fold (Table 2), 11 genes were present at higher levels in the IF library, which were associated with defence (2), metabolism (6), signal transduction (2) and transport (1). The greatest differences between IF and PF DEGs were the *DCL3* (dicer-like 3) gene and the vacuolar ATPase subunit F family protein gene, both of which were present 8.95-fold higher in the IF library than in the PF library.

Forty-nine DEGs were less abundant in the IF library. Those present five-fold or more in the PF library are also listed in Table 2, in which 43 genes were classified as defence (5), development (2), metabolism (27), signal transduction (3), transcription (2) and transport (4). The highest DEG was the *FAD5* (fatty acid desaturase 5) gene, which was present at levels 14.82-fold higher than PF levels.

### Real-time RT-PCR analysis

**Figure 9:**
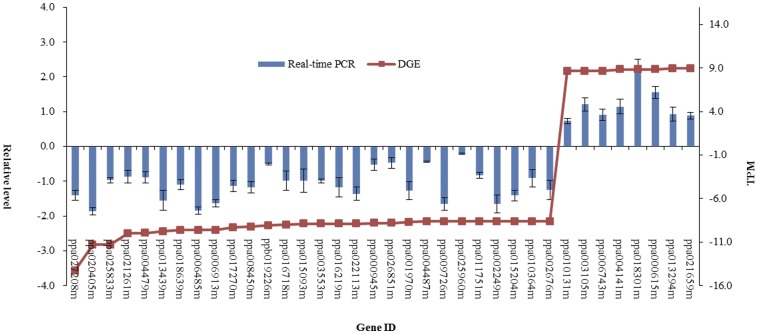
Digital gene expression tag profiling and quantitative real-time PCR analysis of the expression of randomly selected genes. All real-time PCR reactions were repeated three times and the data are presented as the mean ± SD. The x-axis indicates the different genes. The y-axis shows the expression levels: the left shows the relative expression level by qRT-PCR and the right shows tag number per million tags by DGE.

To confirm the reliability of Illumina sequencing technology, 36 genes were randomly selected for quantitative RT-PCR assays. The actual melting curves of 36 genes are showed in the Table S2. The expression level of each gene in the perfect and imperfect flower buds was compared with its abundance from the sequencing data of DGE sequencing (Figure 9). The apparent discrepancies with respect to ratio can be attributed to the essentially different algorithms determined by the two techniques. In the analysis of gene profiles, the DGE method generates absolute rather than relative expression measurements. However, the results showed that expression of these 36 genes was consistent between the qRT-PCR and the DGE analyses. Transcripts from highly abundant Illumina tags appeared at the expected high expression level in the quantitative PCR analyses. Additionally, high-fold changes were observed for genes that showed low copy-numbers in the PF library but high abundances in the IF library. For example, *CPK13* (calcium-dependent protein kinase 13; ppa004141m) showed no expression in the PF library, whereas it was detected at levels of 8.86-fold in the IF library. It was significantly up-regulated by more than 15-fold in the RT-PCR analysis. Similarly, *DCL3* (ppa021659m) was induced by more than 5-fold. In addition, *FAD5* (ppa027208m) showed no expression in the IF library, while it was detected 14.26-fold in the IF library, and was significantly down-regulated in the RT-PCR analysis. These results confirmed the reliability of the transcriptome analysis.

## Discussion

Illumina sequencing technology, a high-throughput sequencing approach, was utilised to estimate gene expression in libraries prepared from perfect and imperfect flower bud tissues. The results (Figure 2) provide estimates of gene expression as determined by the frequency that any given tag (representing a transcript) is sequenced. The data indicate that there is sufficient coverage depth to reach saturation, i.e. there is a complete assessment of all transcripts present in the libraries. This study demonstrated differential transcript abundance and regulation in the development of pistils in Japanese apricot between perfect flower buds and imperfect flower buds. The phenomenon of imperfect flowers widely occurs and seriously affects the production yield; therefore it is necessary to identify the genes related to the molecular mechanism of pistil abortion in the Japanese apricot. These results represent the first large-scale investigation of gene expression in the pistil abortion analysis of Japanese apricot. 17,056 putative genes were identified among the Illumina sequencing tags for the PF library and 16,386 were identified for the IF library. The steady-state transcript level for a set of selected genes was confirmed by real-time (RT)-PCR. Although the differences in gene expression did not match the magnitude of those detected by the Solexa-based sequencing method, the trends of up- and down-regulation were similar. The lower expression level detected by RT-PCR could be due to the difference in sensitivity between the two technologies [37]. Solexa sequencing has been documented to be more sensitive for the estimation of gene expression, especially for low-abundance transcripts, compared to microarrays and real-time RT-PCR [38].

60 DEGs were found to have differences greater than five-fold (Table 2), 54 of which were associated with metabolism (33), defence (7), signal transduction (5), transport (5), development (2) and transcription (2). These transcripts possibly encode genes responding for pistil abortion or programmed cell death (PCD), which were broadly grouped into the following categories based on their known roles in other plant systems:

### Defence response genes

There were two up-regulated genes and five down-regulated genes in the defence response group. Among the defence response genes, *CPK13* [39], CPR30 F-box and associated interaction domains-containing protein [40], *ENODL5* (early nodulin-like protein 5) [41], *FRO2* (ferric reduction oxidase 2) [42], [43], and *MAPKKK15* (mitogen-activated protein kinase kinase kinase 15) [44] have been widely studied in plant pathogen resistance. The late embryogenesis abundant protein (LEA) family protein showed less expression in the imperfect flower buds, which was consistent with previous research on the comparative proteomic analysis of pistil abortion in Japanese apricot and belonged to the group of down-regulated genes [45]. This was similar to many other cold resistant proteins [46], a number of which enhance freezing tolerance by putatively stabilising membranes, or increasing levels of protective osmolytes [8]. A study on chickpeas showed reduced ovule size, reduced ovule viability, missing embryo sacs, and impaired pistil function in temperature-sensitive cultivars for cold stress [47]. In addition, LEA proteins contain hydrophilic side-chains thought to interact with and protect hydrophilic proteins during desiccation by causing protein aggregation [48], [49]. Water stress imposed during the development of flowers is a major factor increasing the rate of abortion, and long-term or frequent water deficits during this period might decrease yield [50], [51]. In our study, LEA protein expression in perfect flower buds was higher than that in imperfect flower buds. Therefore, we speculate the reduction of this temperature-resistant protein might increase the ratio of pistil abortion. There were both up-regulated and down-regulated genes in this category. Temperature and water stress response-related proteins were associated with hot or cold temperature stresses and may also play important roles in pistil abortion in Japanese apricot.

### Genes involved in RNA metabolism

In our study, there were six up-regulated genes and down-regulated genes involved in different kinds of metabolism, such as RNA, protein, pectin and fatty acid metabolism.

The EMB3011 RNA helicase family protein [52], peptidyl-tRNA hydrolase family protein [53], *DCL3* [54], [55] and pre-mRNA-splicing factor CWC26 [56], [57] are involved in RNA processing, including small RNA metabolism. DCL3 produces 24-nt small interfering RNAs (siRNAs) at heterochromatic loci [58]. Plants can utilise siRNAs to guide *de novo* DNA methyltransferases for the establishment of sequence-specific DNA methylation [59], which has several functions in angiosperms, such as transposon silencing and transcriptional gene silencing (TGS) [60]. In *Arabidopsis thaliana*, double mutants between *dicer-like 1* and *dicer-like 3* exhibit a delay in flowering that is caused by increased expression of the floral repressor *FLOWERING LOCUS C* [61]. This delayed-flowering phenotype is similar to that of autonomous-pathway mutants, and the flowering delay can be overcome by vernalisation [61], [62]. In fact, small RNAs play important roles in the termination of floral stem cells, especially miR172 and miR165/166[63]. We have found that miR319, miR319a, miR319e, miR160, miR393, miR394 are significantly differentially-expressed between perfect and imperfect flower buds, and that most of their targets were transcription factors or F-box proteins [64]. In our study, the genes involved in RNA metabolism were up-regulated in pistil abortion, and their expression was higher in the imperfect flower buds than that in the perfect flower buds. These data provide clues to the molecular mechanism of pistil abortion regulated by small RNA metabolism in Japanese apricot.

### Cell-wall organisation: pectin and fatty acid metabolism

In this study, we have found many genes related to cell-wall organisation. Peptidyl-tRNA hydrolase has connections to cell-wall carbohydrates and morphogenesis and belongs to a highly interconnected core network of G-protein signalling [65]. LTP3 is involved in cutin synthesis during the fibre primary cell wall synthesis stage [66].

XTHs are cell-wall enzymes that catalyse the cleavage and molecular grafting of xyloglucan chain functions in the loosening and rearrangement of the cell wall [67], [68]. In our previous study, we found that XTH2 expression was higher in the perfect flower buds than that in the imperfect flower buds [45]. As they are involved in the modification of the load-bearing cell-wall components, they are believed to be very important in the regulation of growth and development [69]. Hyodo et al. (2003) showed that XTH9 tends to be expressed strongly in rapidly dividing and expanding tissues in *Arabidopsis* [68].

Plant invertase/pectin methylesterase inhibitor superfamily protein and PPME1 pectin lyase-like superfamily protein, two down-regulated genes, are involved in pectin metabolism. The homogalacturonan (HGA) fraction, a major component of pectins, is typically deposited into the apoplasm in a highly methylesterified condition. Pectin methylesterase in the apoplasm catalyses the demethylesterification of these HGAs, exposing carboxyl residues, which can be cross-linked by calcium [70]. These changes significantly affect the rheological properties of the cell wall as well as its porosity and ionic status. It follows that the tight control of PME activity, both spatially and temporally, occupies a pivotal position in the control of cell-wall growth and development [71]. An important post-transcriptional mechanism of regulation of these enzymes is represented by proteinaceous inhibitors [72]. Our previous research found that the pistil of imperfect flowers stopped differentiation in early December and finally disintegrated, while the pistil of perfect flowers continued to differentiate and developed perfectly (Shi et al., 2011). This phenomenon might result from the strain formation of cell walls.

*FAB1* [73] and *FAD5* [74] are involved in unsaturated fatty acid biosynthesis. Unsaturated fatty acid plays a very important role in lipid biosynthesis and cell wall organisation. These two genes were down-regulated in pistil abortion, and their expression was higher in the perfect flower buds than in the imperfect flower buds. Therefore, we hypothesise that the forming of an imperfect flower has a crucial relationship with the cell-wall organisation.

## Materials and Methods

### Plant materials

The ratio of imperfect flowers of Japanese apricot cultivars Daqiandi is about 76%. While the pistil of a perfect flower continues to differentiate and develop perfectly, the pistil of an imperfect flower stops differentiation in early December and finally disintegrates. We chose two types of flower buds of this period from Daqiandi trees grown in the National Field GenBank for Japanese Apricot, Nanjing, Jiangsu Province, China. All of the samples were collected and immediately frozen in liquid nitrogen and stored at −80°C.

### DGE library construction and Illumina sequencing

To construct DGE libraries, total RNA was extracted from perfect and imperfect flower buds using the method according to Meisel *et al*. [75]. Library construction was carried out at the Beijing Genomics Institute (BGI, Shenzhen, Guangdong, China), using Illumina's DGE tag profiling technology. The main reagents and supplies were the Illumina Gene Expression Sample Prep Kit and the Solexa Sequencing Chip (flowcell), and the main instruments were the Illumina Cluster Station and the Illumina HiSeq™ 2000 System. 6 µg of total RNA was extracted, oligo(dT) magnetic bead adsorption was used to purify mRNA, and then oligo(dT)s were used as primers to synthesise the first and second-strand cDNA. The 5′ ends of tags could be generated by two types of endonuclease: *Nla*III or *Dpn*II. The bead-bound cDNA was subsequently digested by the restriction enzyme *Nla*III, which recognised and cut the CATG sites. The fragments (except for the 3′ cDNA fragments) connected to oligo(dT) beads were washed away and the Illumina adaptor 1 was ligated to the sticky 5′ end of the digested bead-bound cDNA fragments. The junction of Illumina adaptor 1 and the CATG site was the recognition site of *Mme*I, which is a type of endonuclease with separated recognition and digestion sites. This enzyme cut 17bp downstream of the CATG site, producing tags with adaptor 1 ends. After removing 3′ fragments with magnetic bead precipitation, the Illumina adaptor 2 was ligated to the 3′ ends of tags, acquiring tags with different adaptors at both ends to form a tag library. After 15 cycles of linear PCR amplification, 105bp fragments were purified by 6% TBE PAGE gel electrophoresis. After denaturation, the single-chain molecules were fixed onto the Illumina Sequencing Chip (flowcell). Each molecule grew into a single-molecule cluster sequencing template through *in situ* amplification, and four types of nucleotides that were labelled with four colours were added, before sequencing was performed using the method of sequencing by synthesis (SBS). Each tunnel generated millions of raw reads with a sequence length of 49bp.

### Bioinformatics analysis of sequencing data

Sequencing-generated raw image data were transformed by base calling into sequence data, which was called raw data or raw reads. Raw sequences had 3′ adaptor fragments as well as a few low-quality sequences and several types of impurities. Raw sequences were transformed into clean tags after the following data-processing: first, the 3′ adaptor sequence was removed, since tags were only 21nt long, but the sequencing reads were 49nt long, raw sequences included the 3′ adaptor sequences; second, empty reads (reads with only 3′ adaptor sequences but no tags) were removed; third, low-quality tags (tags with unknown sequences 'N') were removed; fourth, tags which were too long or too short were removed, leaving tags of 21nt long; and lastly, tags with a copy number of 1 (probably a sequencing error) were removed. Then we analysed the clean tag data, including the sequence quality assessment, the saturation analysis of sequencing, the experimental repeatability analysis, the distribution of clean tag copy number, and the alignment statistics of clean tags. The saturation analysis can be performed to check whether the number of detected genes increases when the sequencing amount (total tag number) increases. Heterogeneity and redundancy are two significant characteristics of mRNA expression. A small number of categories of mRNA have very high abundance, while the majority remain at a very low level of expression. The distribution of clean tag expression can be used to evaluate the normality of the whole data.

### Gene expression annotation and normalisation

A virtual library was made containing all of the possible CATG+17 bases length sequences of the reference gene sequences (mapped to the peach genome, GDR, http://www.rosaceae.org/species/prunus/peach). All clean tags were mapped to the reference sequences and only 1bp mismatch was considered. Clean tags mapped to reference sequences from multiple genes were filtered. The remaining clean tags were designated as unambiguous clean tags. The number of unambiguous clean tags for each gene was calculated and then normalised to TPM (number of transcripts per million clean tags) [38], [76]. Sense-antisense plays an important role in gene expression regulation. Sequencing tags mapped to the complementary strand of the sense gene suggested that its antisense strand also had transcripts, and this gene may use the sense-antisense regulation. Therefore, clean tags and their antisense were aligned.

### Screening of differentially expressed genes

Regarding the significance of digital gene expression profiles [77], we used a rigorous algorithm supplied from BGI to identify differentially-expressed genes between two samples [26]. The *P*-value corresponds to differential gene expression. FDR (False Discovery Rate) is a method to determine the threshold of the *P* value in multiple tests and analyses through manipulating the FDR value. For example, assume that we have picked out R differentially-expressed genes in which S genes really show differential expression and the other V genes are false positives. If we decide that the error ratio is ‘Q = V/R’ must stay below a cut-off (e.g. 1%), we should pre-set the FDR to a number no larger than 0.01 [78]. We use FDR≤0.001 and the absolute value of |log2Ratio|≥1 as the threshold to judge the significance of gene expression difference. More stringent criteria with smaller FDR and a bigger fold-change value can be used to identify differentially expressed genes.

### Functional analysis

To determine the main biological functions, DGE were mapped to every node of the Gene Ontology (GO) database (http://www.geneontology.org/) and the pathway enrichment was analysed. Gene Ontology is an international standardised gene functional classification system that offers a dynamically updated controlled vocabulary and a strictly defined concept to comprehensively describe properties of genes and their products in any organism. GO has three ontologies: molecular function, cellular component and biological process. The basic unit of GO is GO-term. Every GO-term belongs to a type of ontology. Our GO functional enrichment analysis also integrates the clustering analysis of expression patterns [24]. In gene expression profiling analysis, GO enrichment analysis of functional significance applies hypergeometric tests to map all differentially-expressed genes to terms in GO database, looking for significantly enriched GO terms in differentially expressed genes (DEGs) comparing to the genome background. The calculating formula is: 
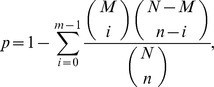
where N is the number of all genes with GO annotation; n is the number of DEGs in N; M is the number of all genes that are annotated to the certain GO terms; m is the number of DEGs in M. The P-value is corrected by Bonferroni, and a corrected P-value ≤0.05 was chosen as the threshold value. The GO term (P≤0.05) is defined as a significantly differentially-expressed gene-enriched GO term. This analysis allowed us to determine the major biological functions of differentially-expressed genes.

Different genes usually cooperate with each other to exercise their biological functions. Pathway-based analysis helps to further understand a gene's biological functions. KEGG is the major public pathway-related database. Pathway enrichment analysis identifies significantly enriched metabolic pathways or signal transduction pathways in DGE compared with the whole genome background. The calculating formula is the same as that in GO analysis. Here, N is the number of genes with a KEGG annotation, n is the number of DEGs in N, M is the number of genes annotated to specific pathways, and m is the number of DEGs in M. The pathways with a Q value of ≤ 0.05 are defined as those with significantly differentially-expressed (enriched) genes. Through the enrichment analysis of DGE pathway significance, the most meaningful pathways can be obtained.

### Verification by real-time quantitative RT-PCR (qRT-PCR)

The same RNA samples were used for these experiments as well as for the DGE, which was isolated according to the method described by Meisel et al. [75]. The genomic DNA contamination was removed with RNase-free DNase I (TaKaRa, Japan) according to the instruction manual. The concentration of the RNA was calculated from the absorbance at 260 nm with a BioPhotometer (Eppendorf, Hamburg, Germany). Purity was verified by an optical density (OD) absorption ratio OD260 nm/OD280 nm between 1.80 and 2.05, and OD260 nm/OD230 nm values ranging from 2.00 to 2.60, while the integrity was evaluated by electrophoresis on ethidium bromide-stained 1.0% agarose gels. Intact rRNA subunits of 18 S and 28 S were observed on the gel and the absence of smears indicated minimal degradation of the RNA.

The first-strand cDNA was synthesised according to the method described by Tong et al. [79]. qPCR was performed using a SYBR® Green real-time PCR Master Mix (TaKaRa, Japan) and all of the primers used and the position information are listed in Table S1 and S2. For each reaction, 1 µL of diluted cDNA (equivalent to 100pg of total RNA) was mixed with 10 µL of 2×SYBR green reaction mix (SYBR® Green qRT-PCR Master Mix; TaKaRa, Japan). 5 pmol of the forward and the reverse primers were added to make a final volume 20 µL. The PCR amplification started with an initial denaturation for 3 min at 95°C, followed by 40 cycles of 95°C for 20 s, 60°C for 20 s, and 72°C for 45 s. The fluorescence signal was measured once every 1°C. Negative PCR controls (no cDNA template) were used to detect possible contamination. The specificity of the primer amplicons was checked by analysis of the melting curve. The RNA polymerase subunit (RP II) was used as a reference gene in the qPCR [79]. Relative gene expression values were calculated using the 2-△△CT method of Ramakers. The data was analysed with an R^2^ above 0.998 using the LinRegPCR program [80].

## Conclusions

We first comparatively constructed the digital gene expression profile between the perfect and imperfect flowers in Japanese apricot. 3,476,249 and 3,580,677 tags were sequenced from two libraries constructed from perfect and imperfect flower buds of Japanese apricot, respectively. There were 689 significantly differentially-expressed genes between the two libraries. GO annotation revealed that highly-ranked genes are those implicated in small molecule metabolism, the cellular component organisation or biogenesis at the cellular level and fatty acid metabolism. The expression patterns of 36 differentially-expressed genes were confirmed by real time RT-PCR. This is the first report on the Illumina RNA-seq technique analysis of differentially-expressed profiles related to pistil abortion that both computationally and experimentally provides valuable information for the further functional characterisation of genes associated with the pistil development in woody plants.

## Supporting Information

Table S1 **The primers designed for qRT-PCR.**
(XLS)

Table S2 **The transcript sequence, primer positions and the melting curves.**
(DOC)
