# Supplementary material for: Identification of Differentially-Expressed Genes Associated with Pistil Abortion in Japanese Apricot by Genome-Wide Transcriptional Analysis
Source: PLoS One. 2012 Oct 16;7(10):e47810. doi: 10.1371/journal.pone.0047810 (PMC3472986; doi:10.1371/journal.pone.0047810)
Supplement: Table S2 — The transcript sequence, primer positions and the melting curves. (DOC) [file pone.0047810.s002.doc]

Table S2. The transcript sequence, primer positions and the melting curves

| The transcript sequence and the primer positions (Arrow section) | melting curves |
| --- | --- |
| 1. ppa027208m |  |
| 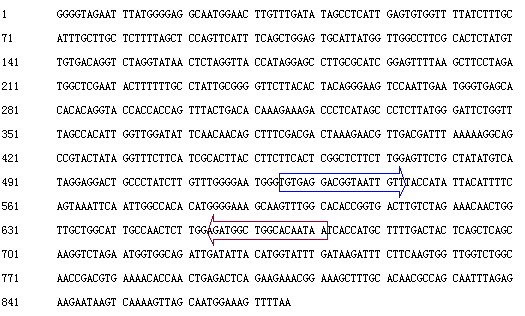 | 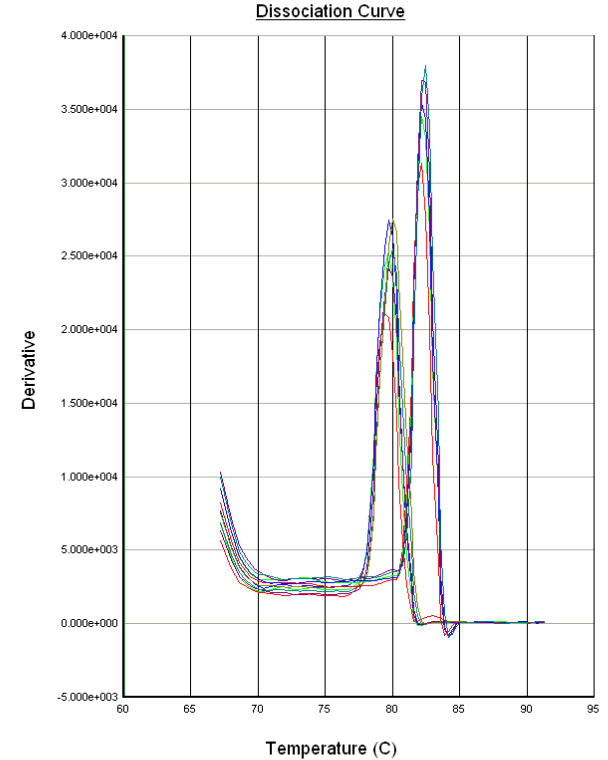 |
| 1. ppa020405m |  |
| 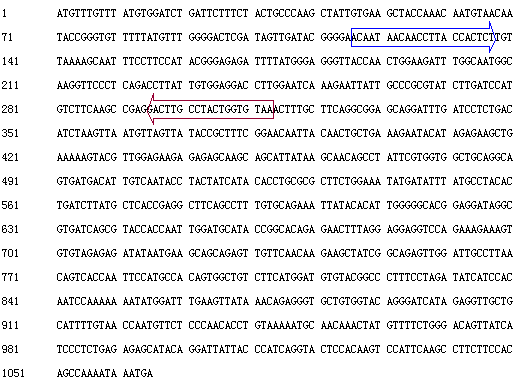 | 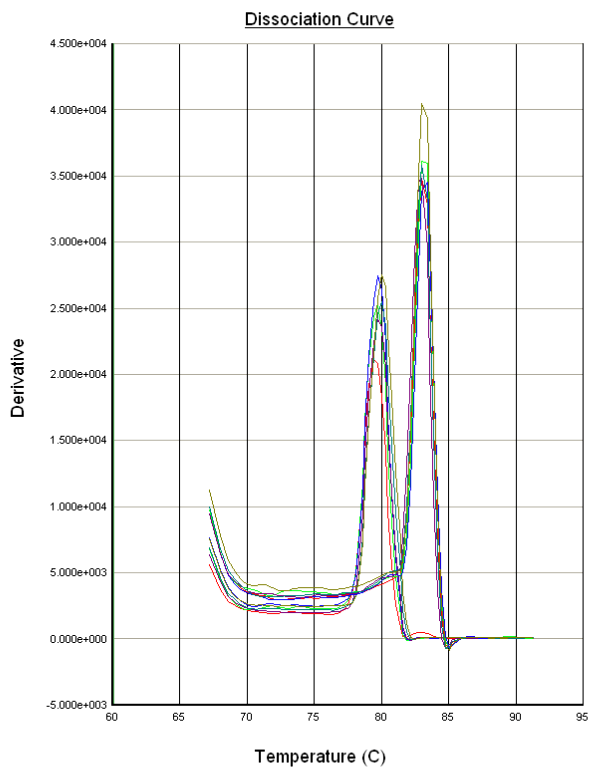 |
| 1. ppa025833m |  |
| 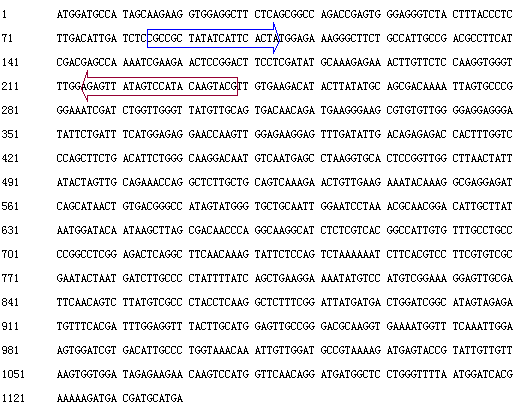 | 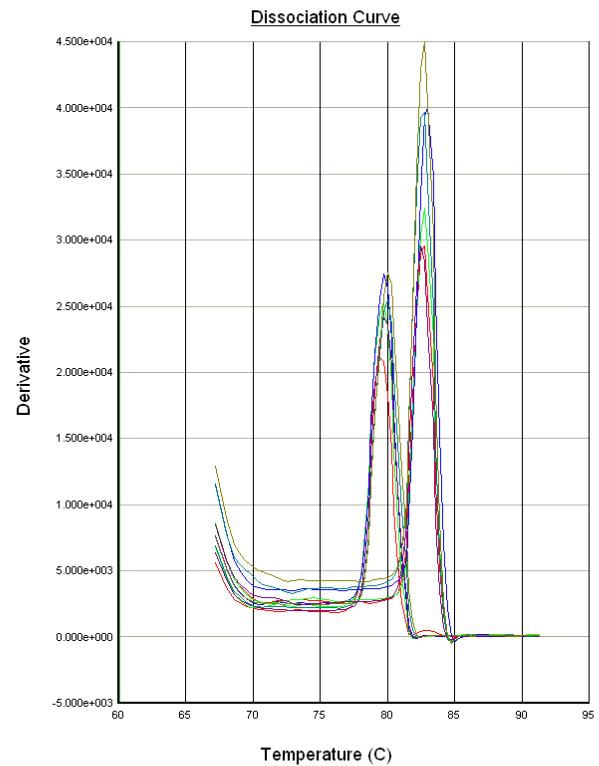 |
| 1. ppa021261m |  |
| 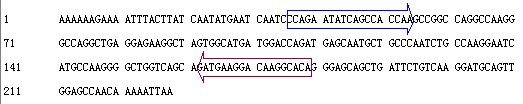 | 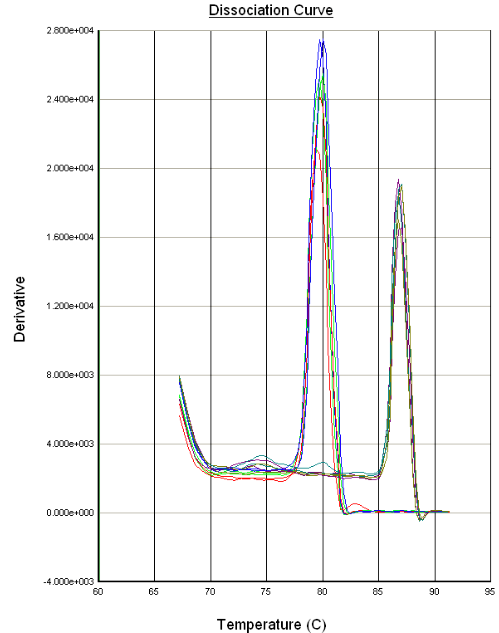 |
| 1. ppa004479m |  |
| 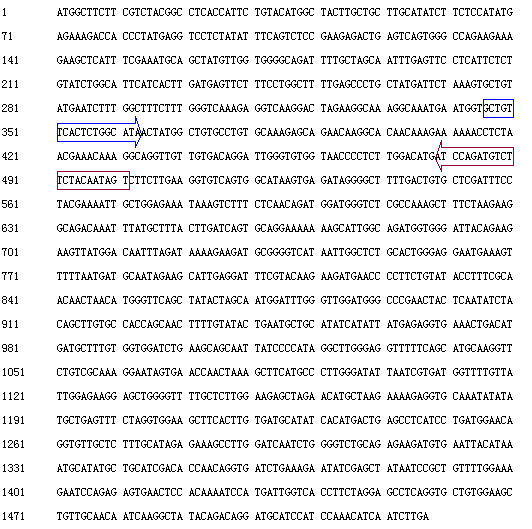 | 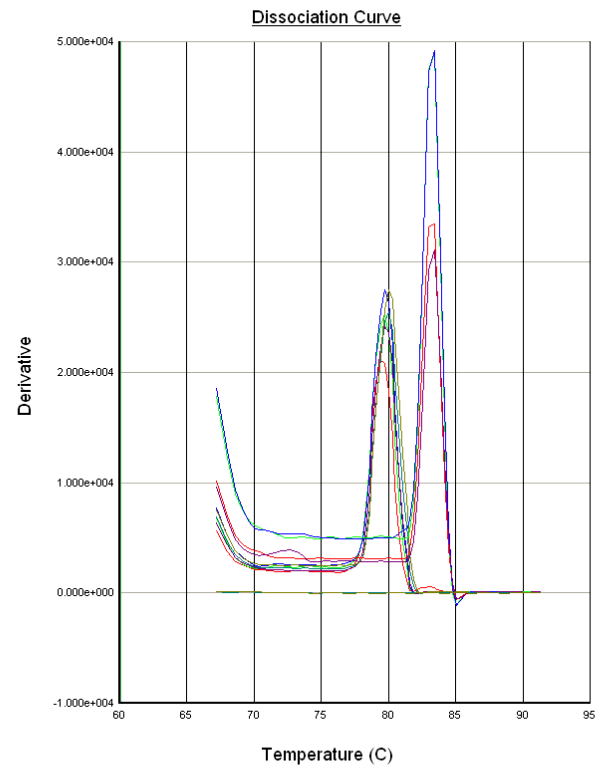 |
| 1. ppa013439m |  |
| 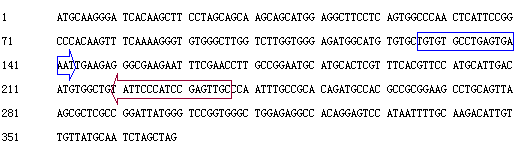 | 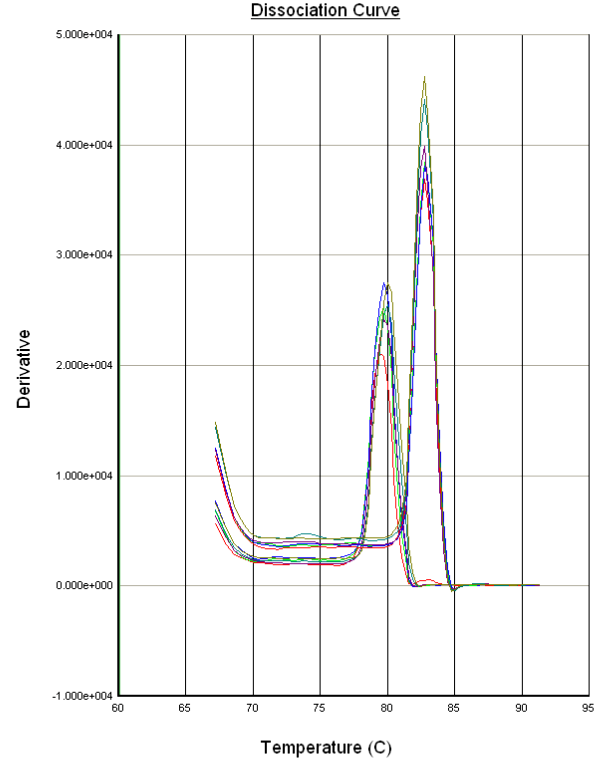 |
| 1. ppa018639m |  |
| 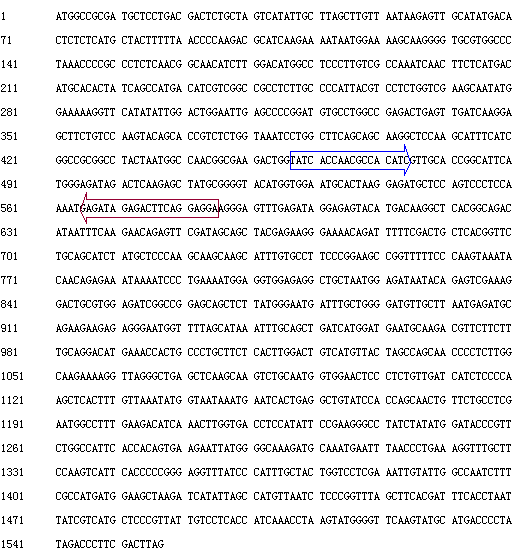 | 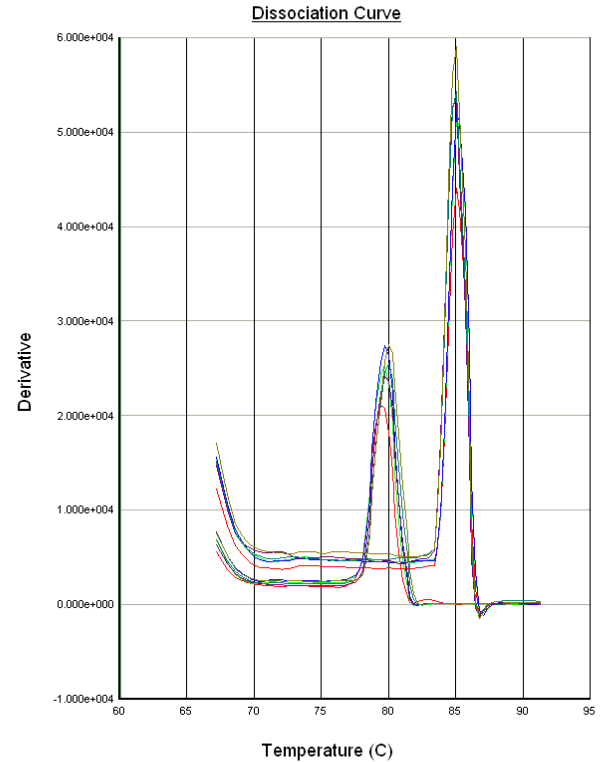 |
| 1. ppa006485m |  |
| 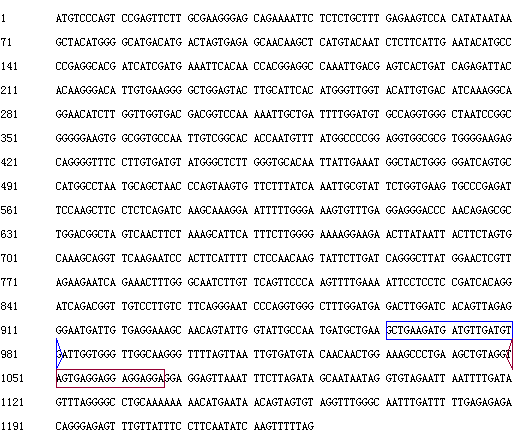 | 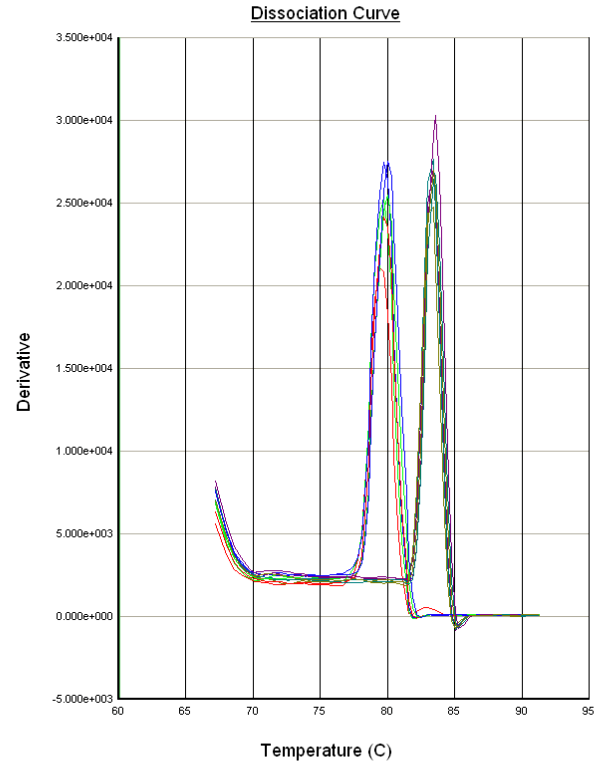 |
| 9. ppa006913m |  |
| 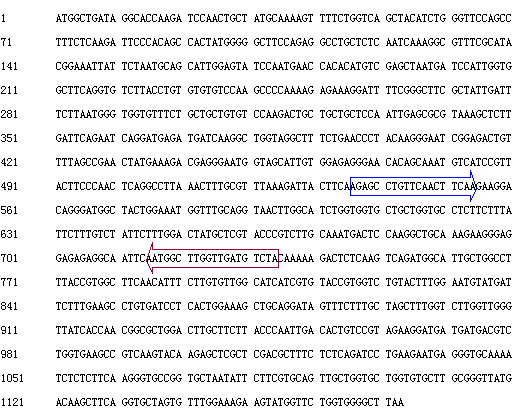 | 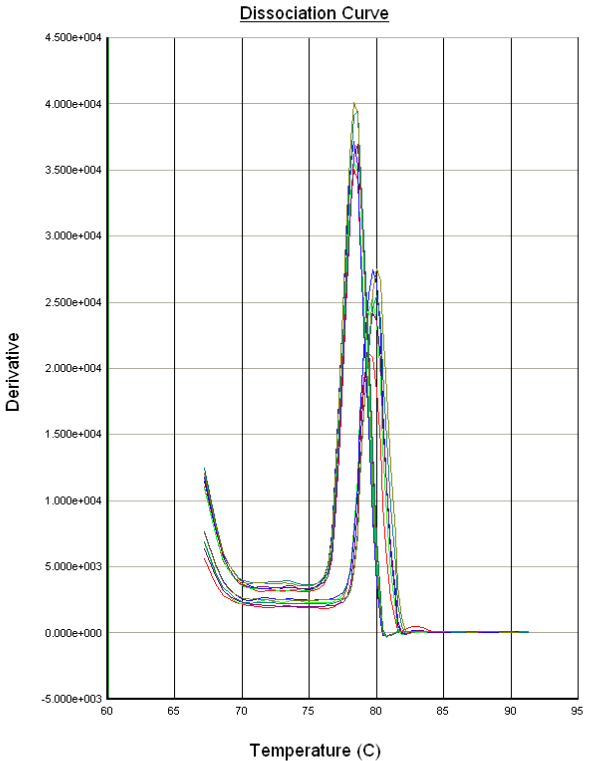 |
| 1. ppa017270m |  |
| 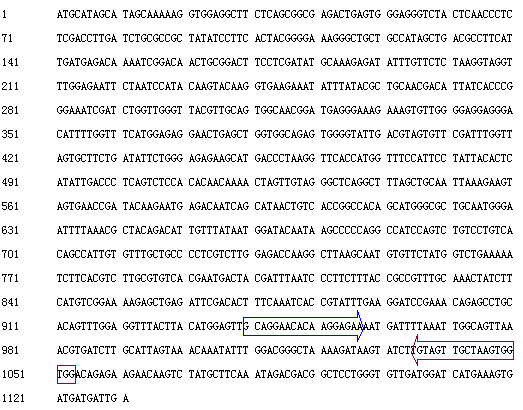 | 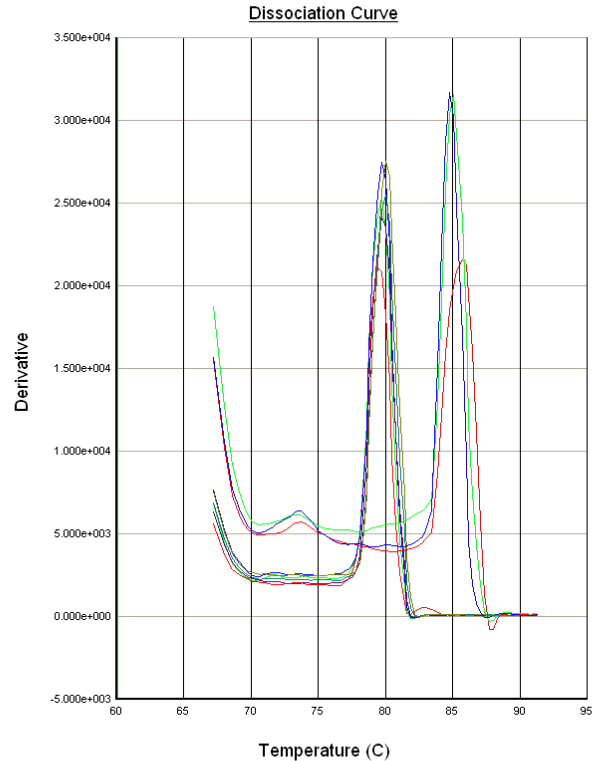 |
| 1. ppa008450m |  |
| 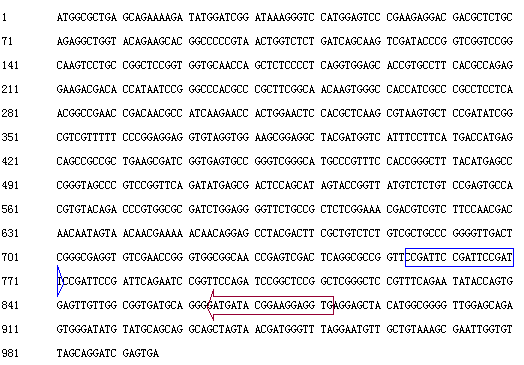 | 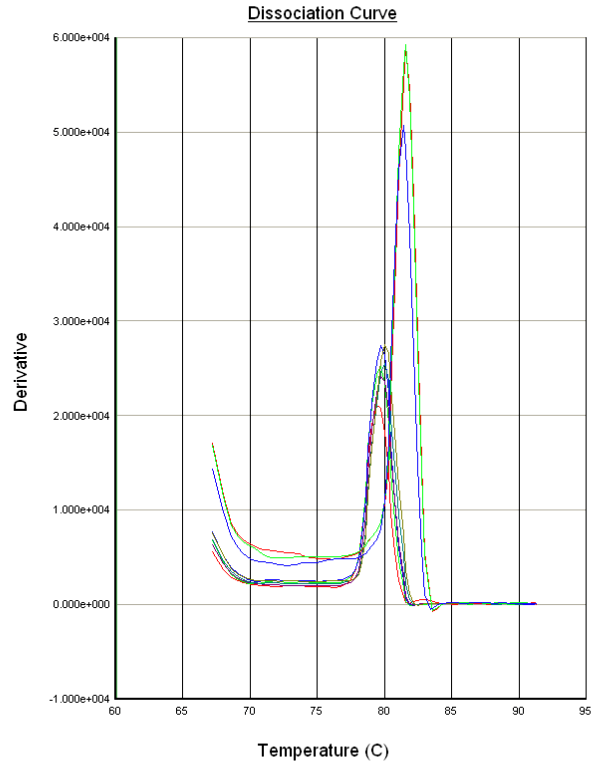 |
| 1. ppb019226m |  |
| 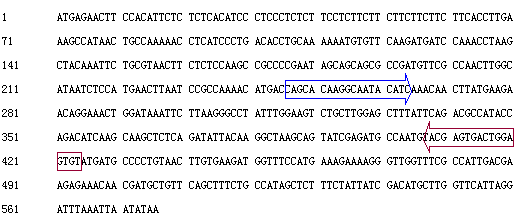 | 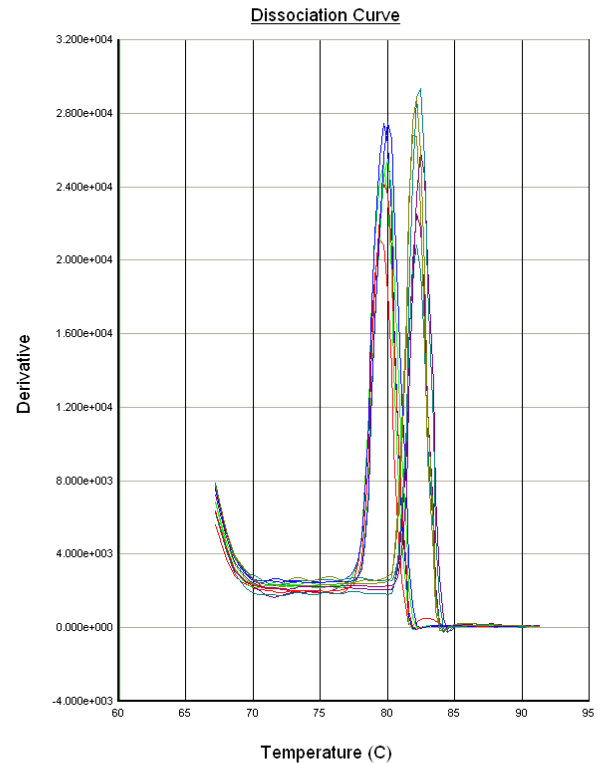 |
| 1. ppa016718m |  |
| 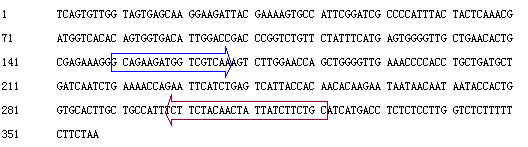 | 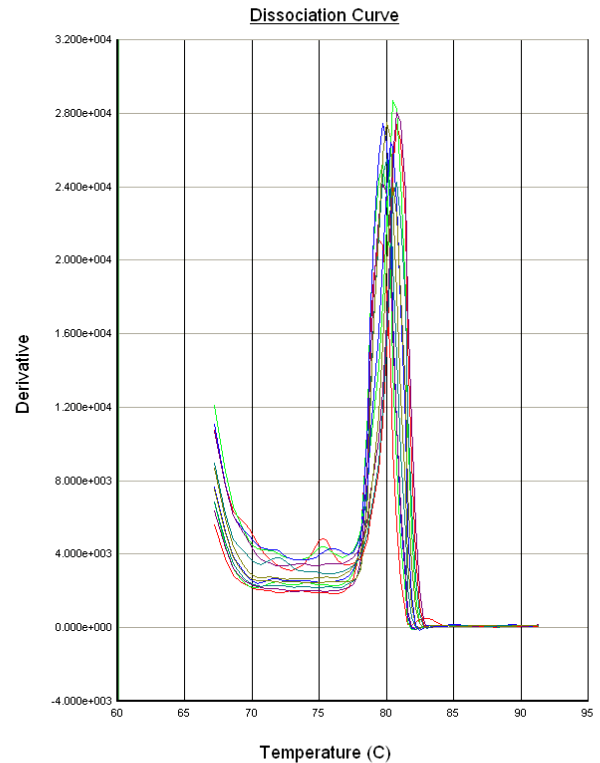 |
| 1. ppa015093m |  |
| 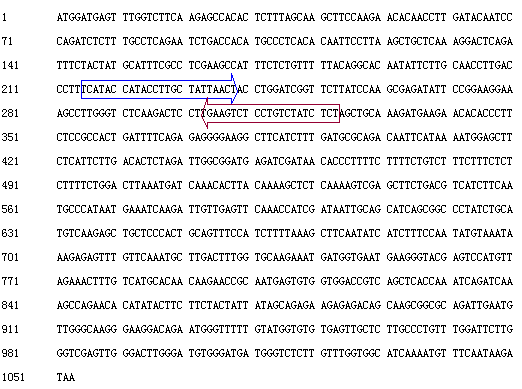 | 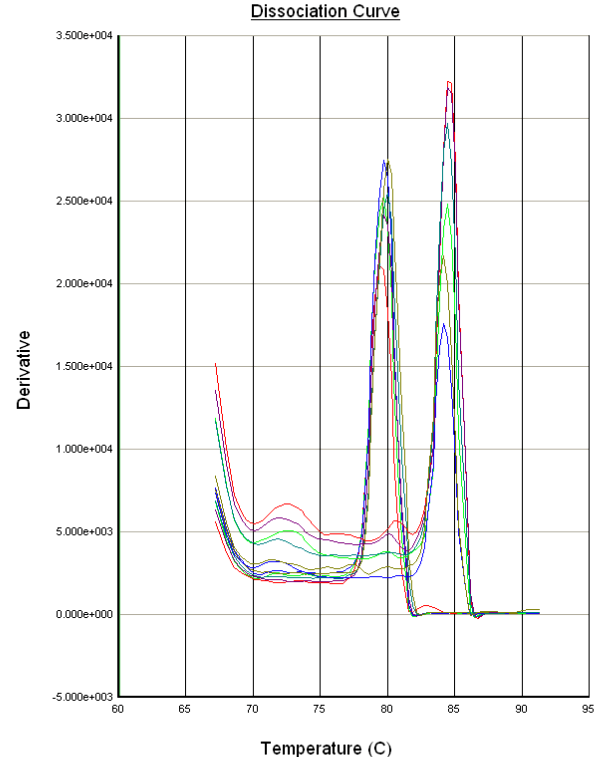 |
| 1. ppa003553m |  |
| 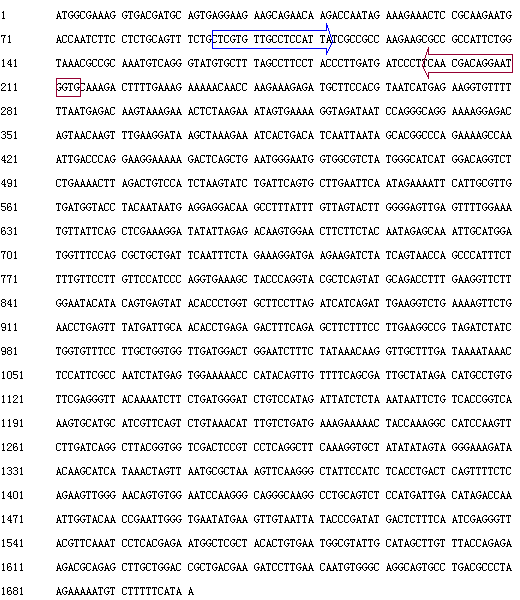 | 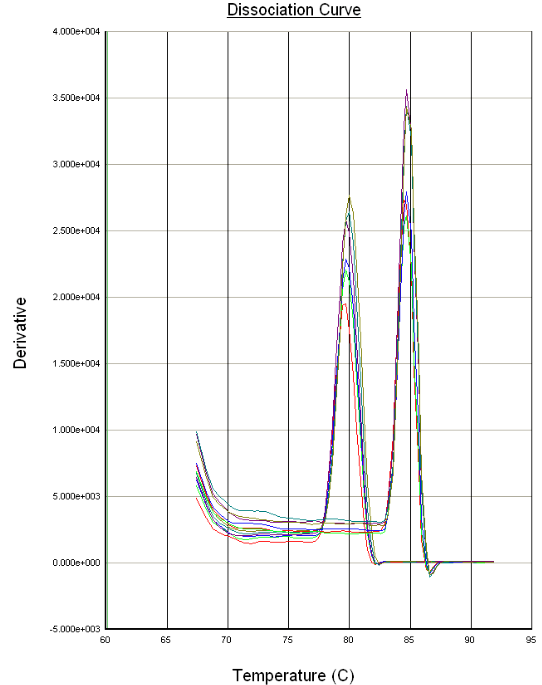 |
| 16. ppa016219m |  |
| 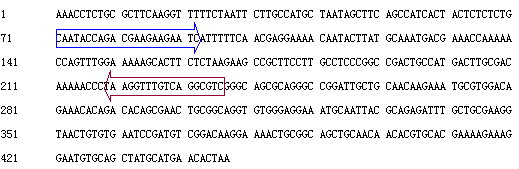 | 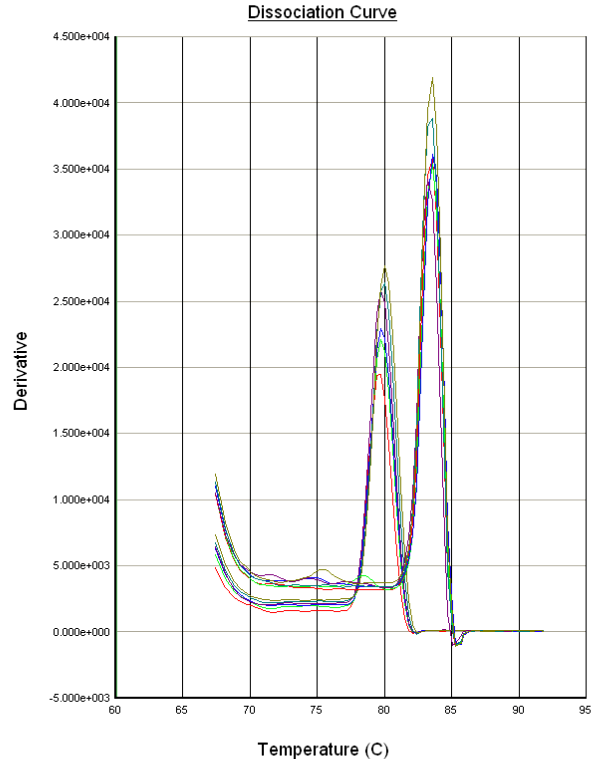 |
| 1. ppa022113m |  |
| 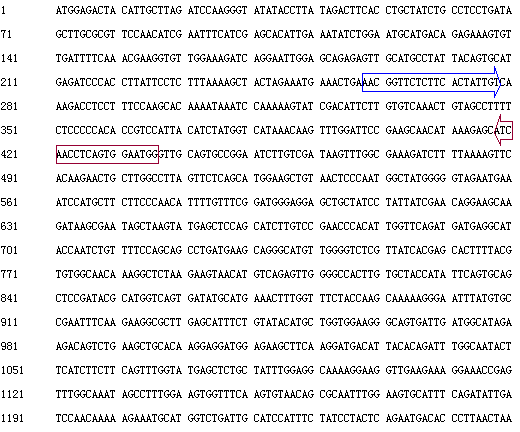 | 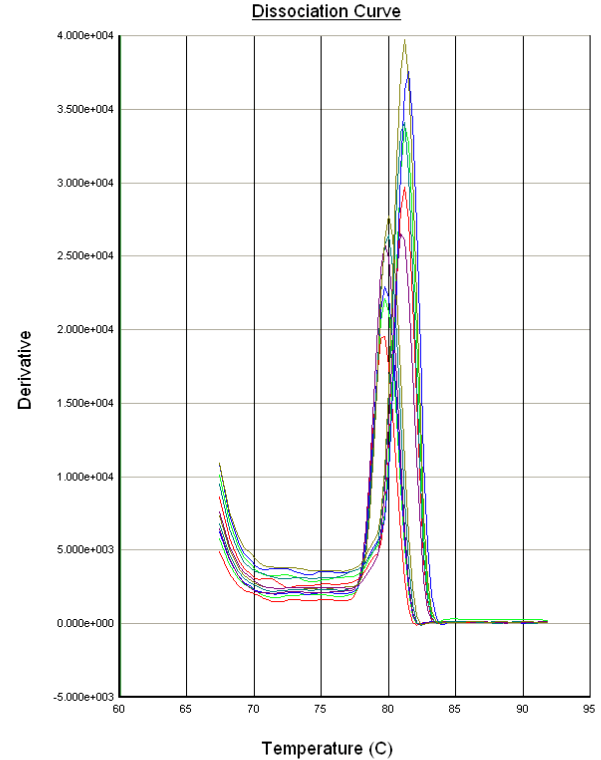 |
| 1. ppa000945m |  |
| 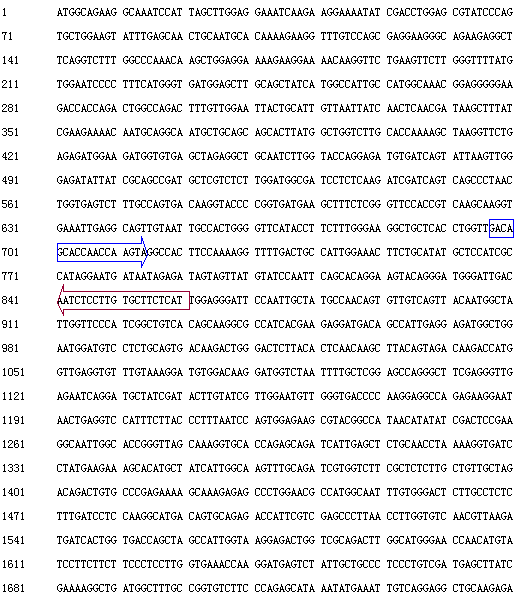  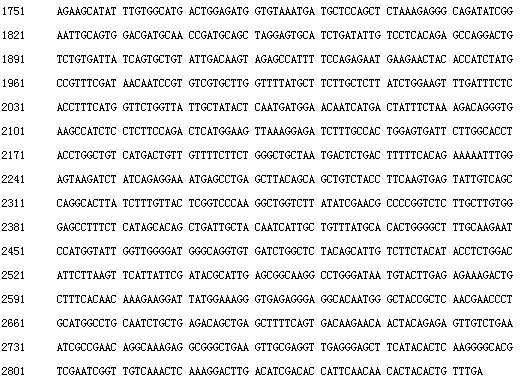 | 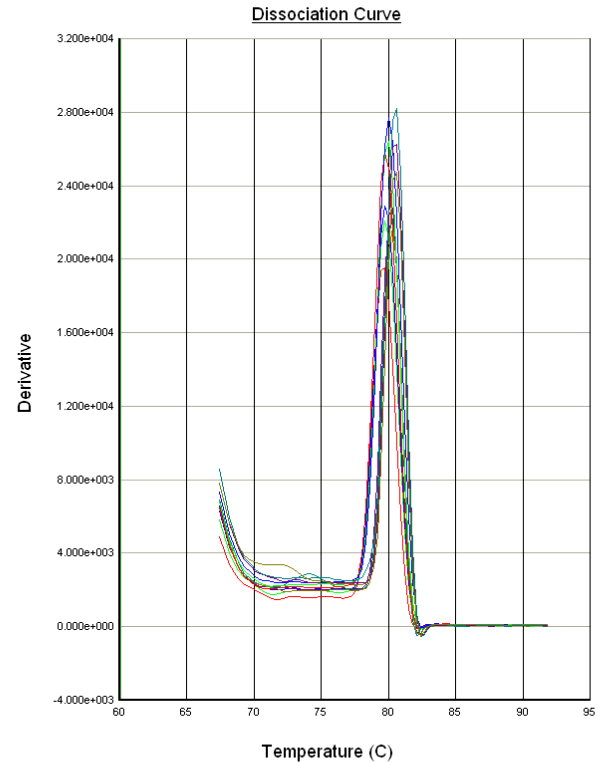 |
| 1. ppa026851m |  |
| 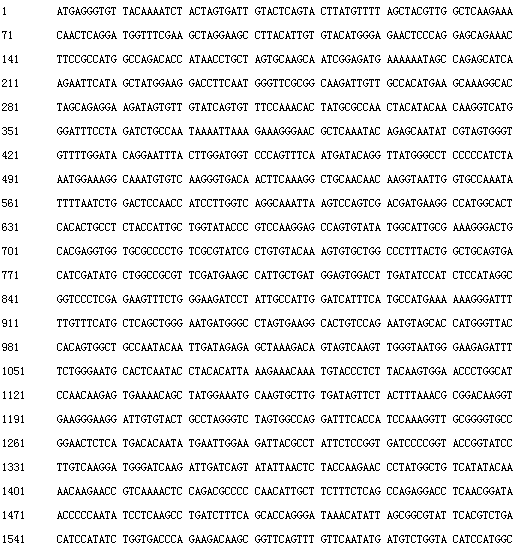  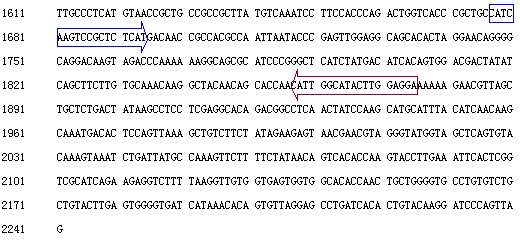 | 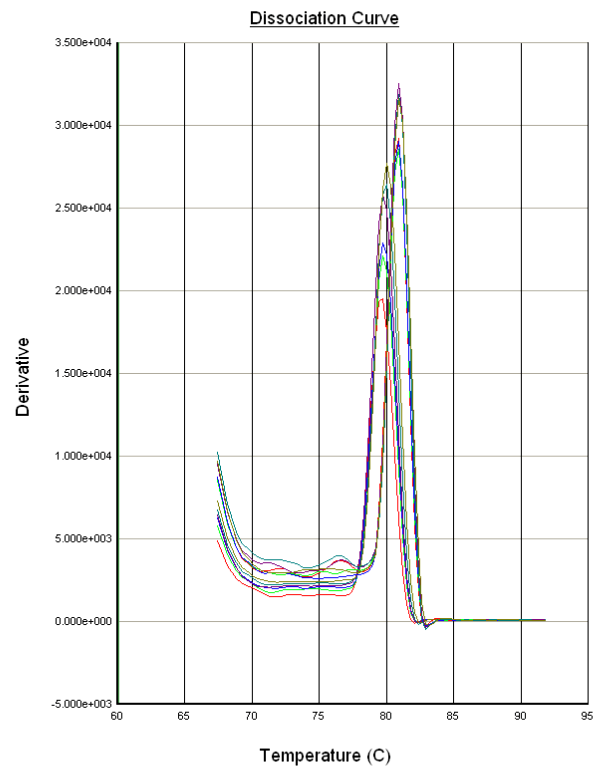 |
| 1. ppa001970m |  |
| 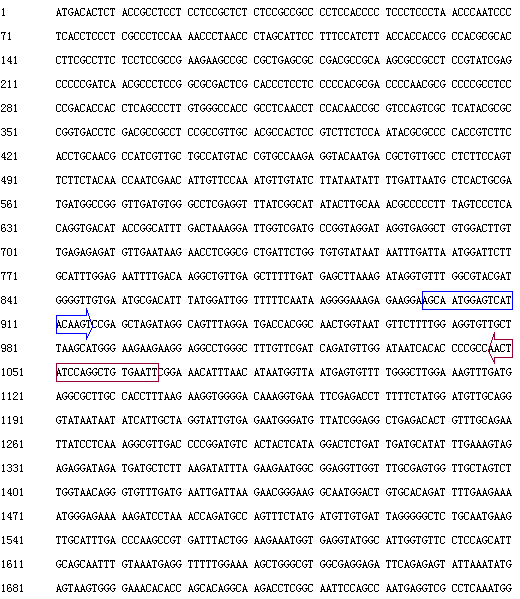  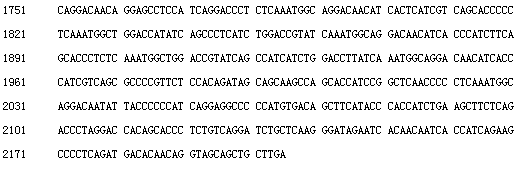 | 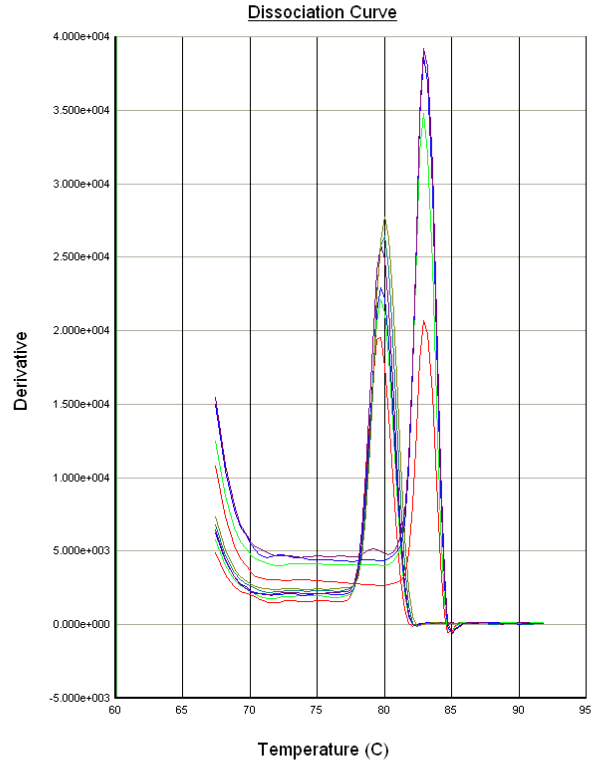 |
| 1. ppa004487m |  |
| 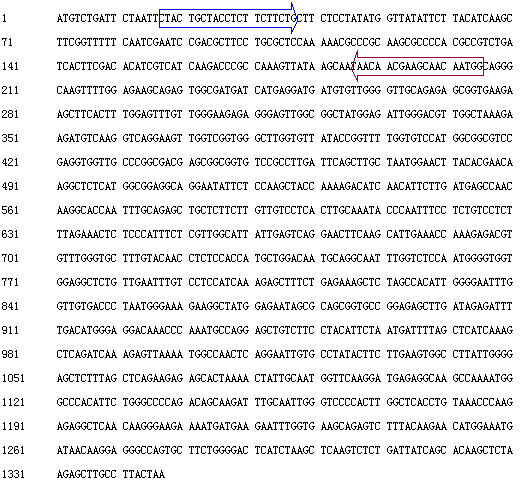 | 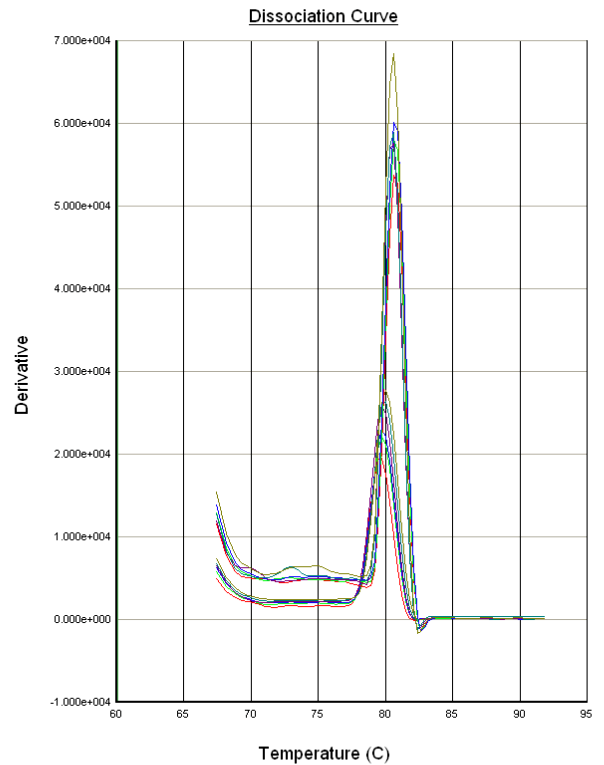 |
| 1. ppa009726m |  |
| 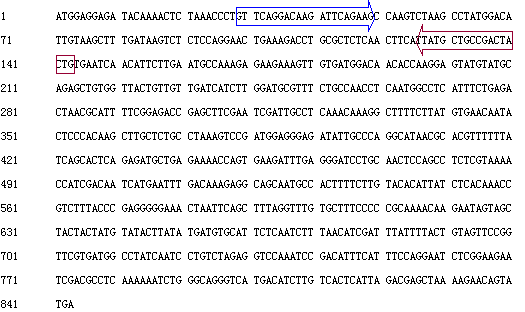 | 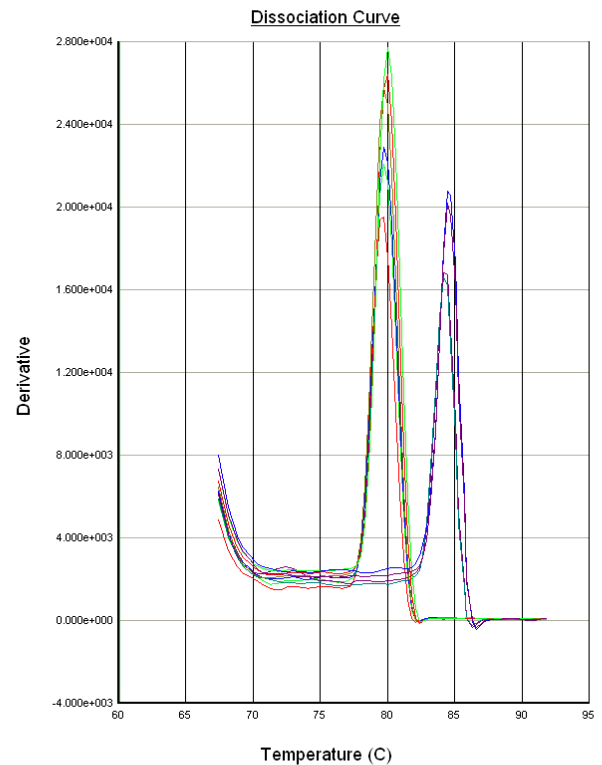 |
| 1. ppa025960m |  |
| 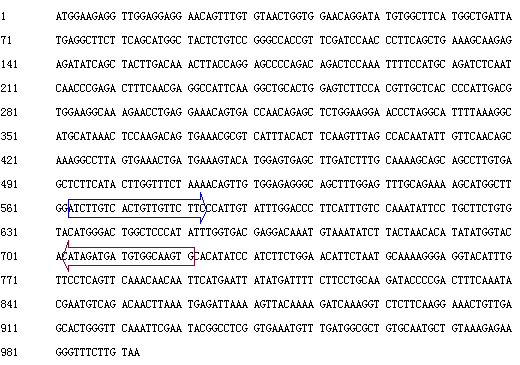 | 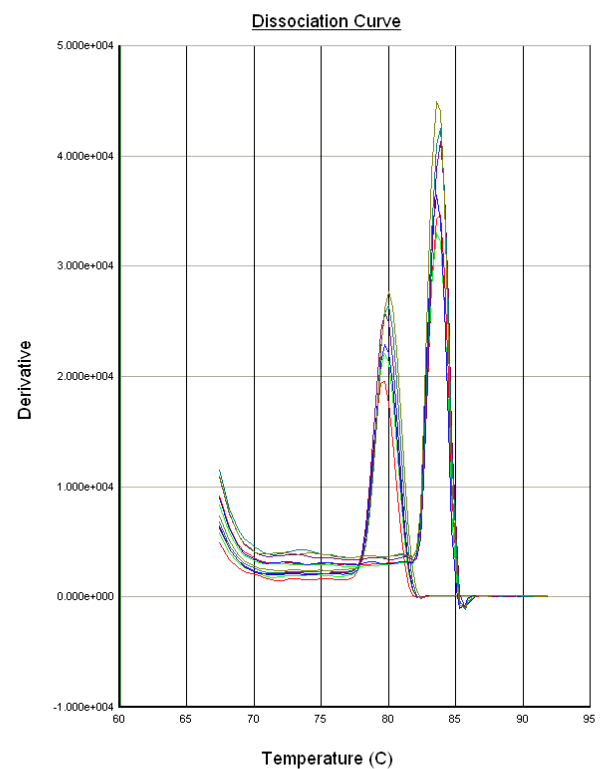 |
| 1. ppa011751m |  |
| 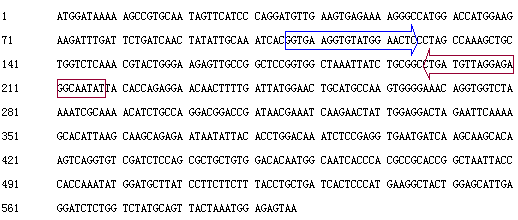 | 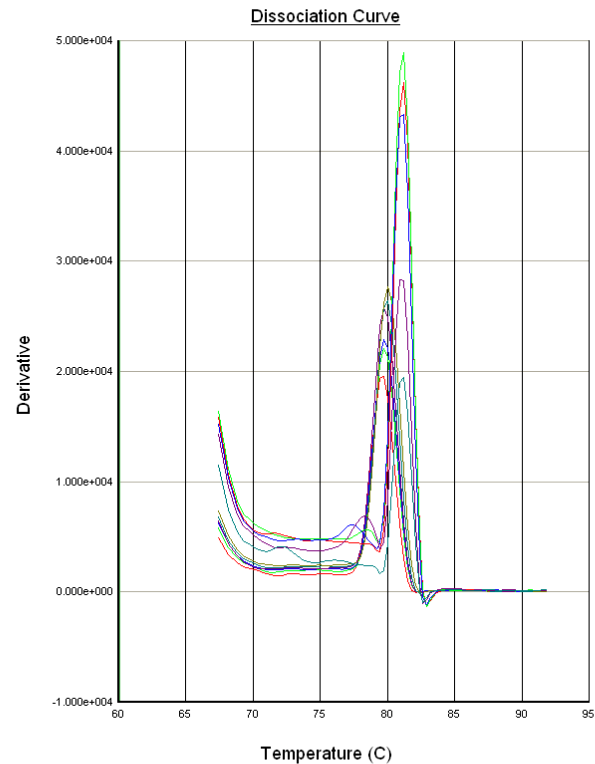 |
| 1. ppa002249m |  |
| 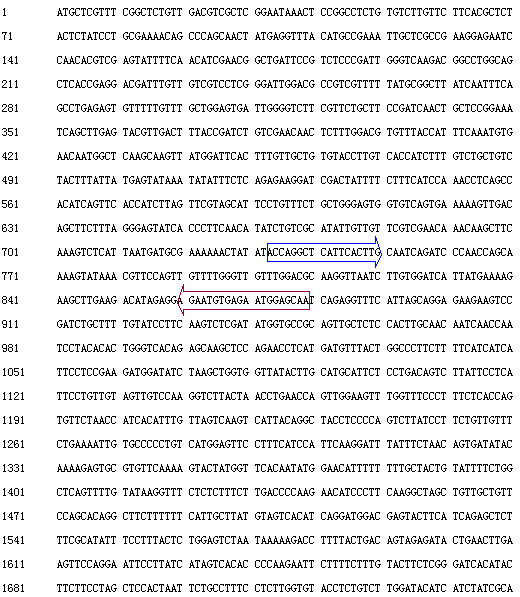  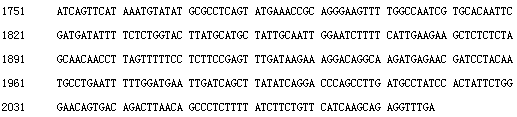 | 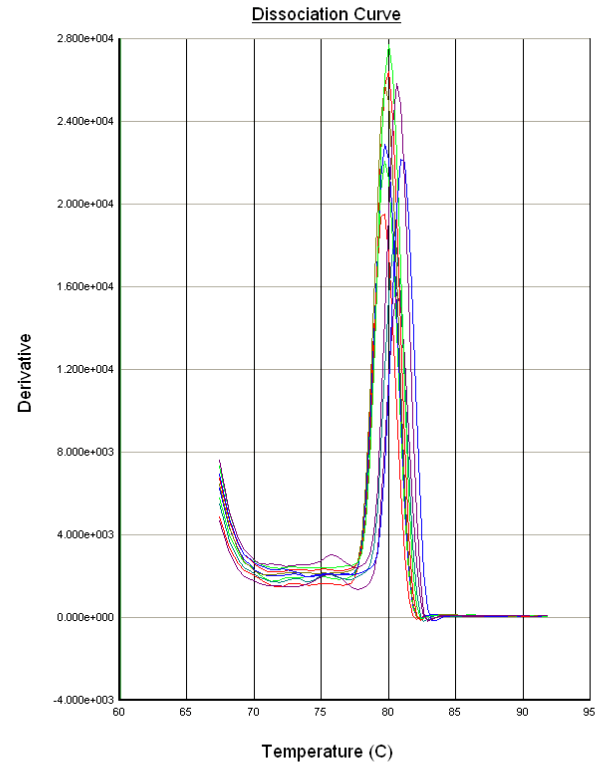 |
| 1. ppa015204m |  |
| 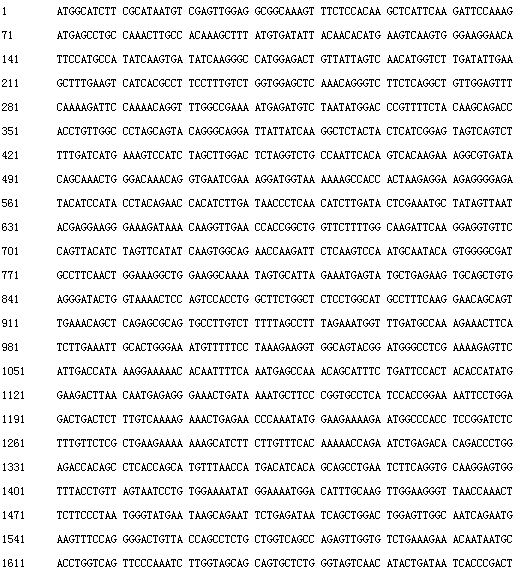  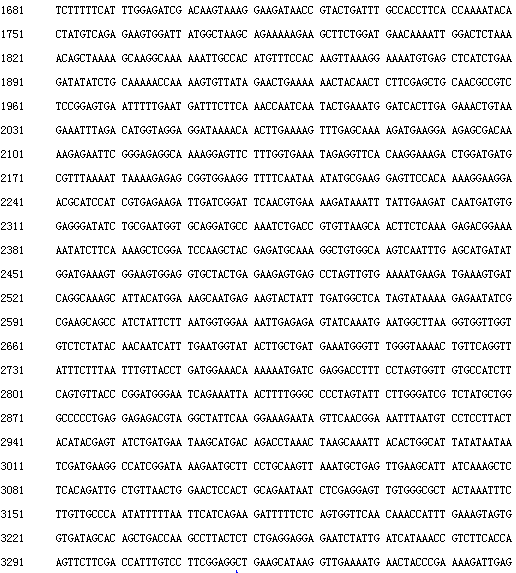  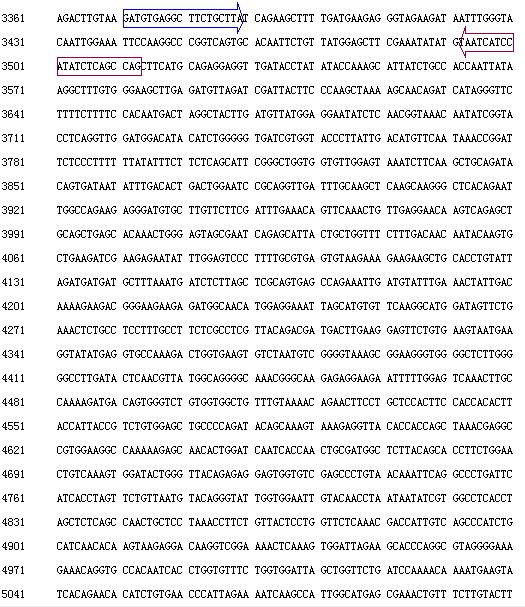  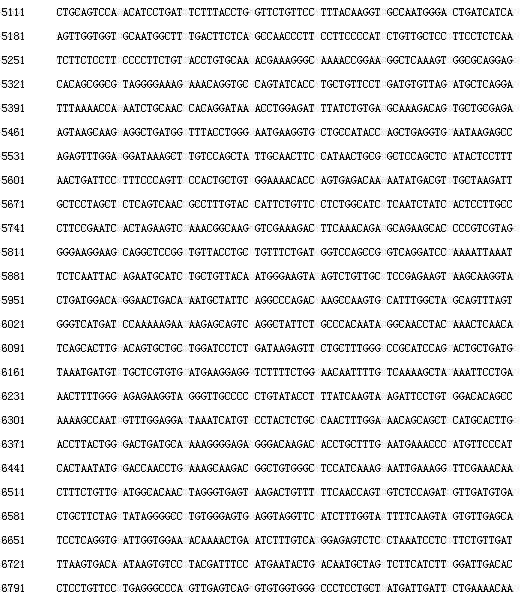  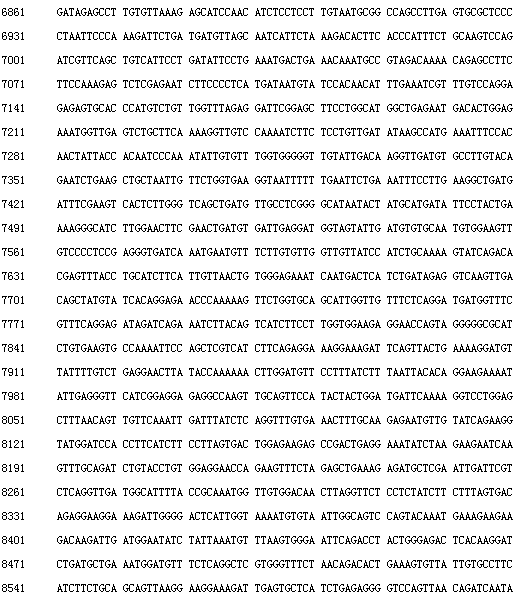  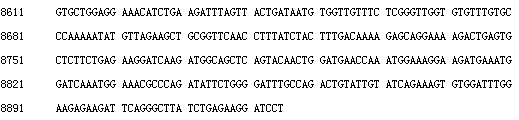 | 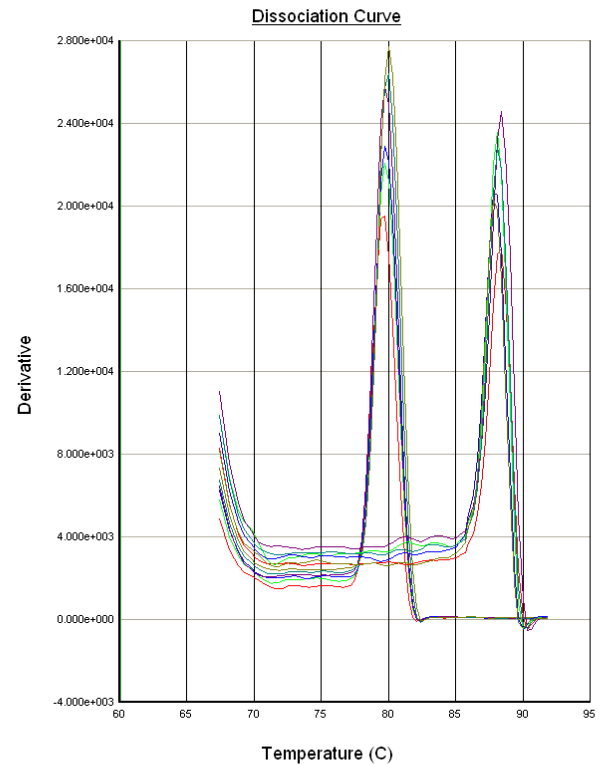 |
| 1. ppa010364m |  |
| 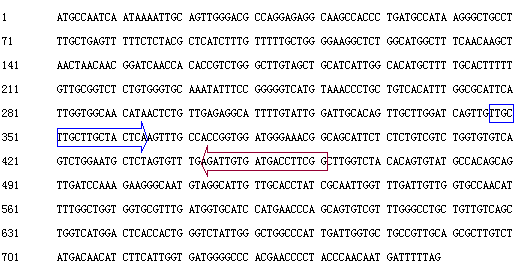 | 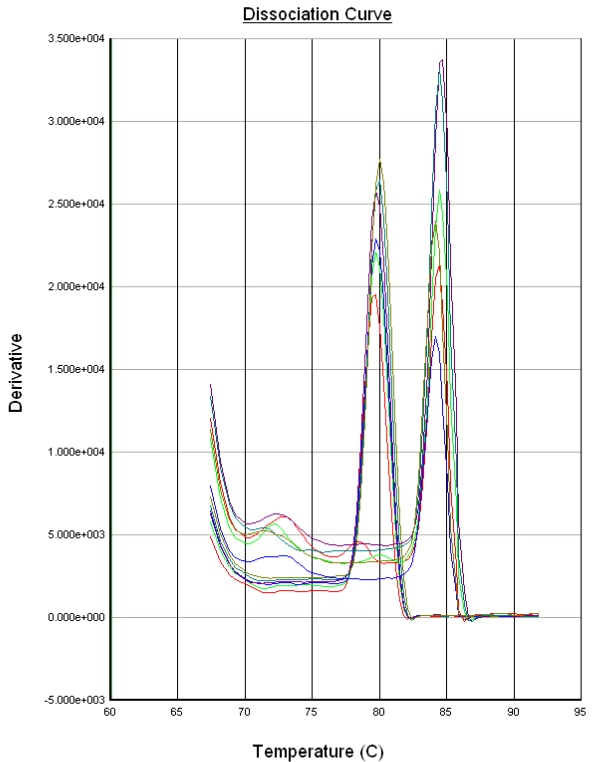 |
| 28. ppa002676m | - |
| 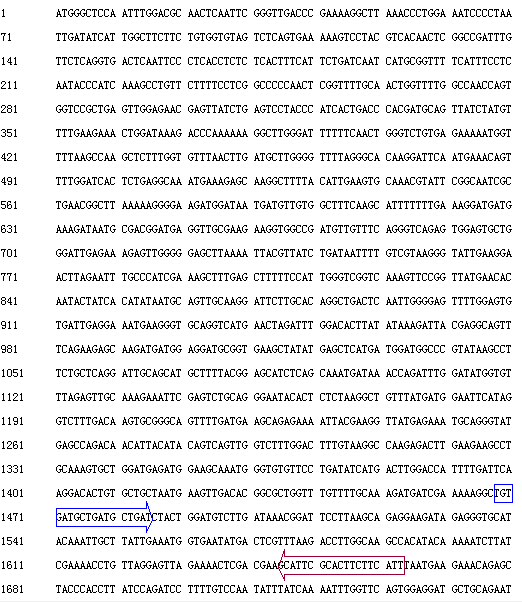  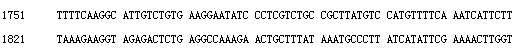 | 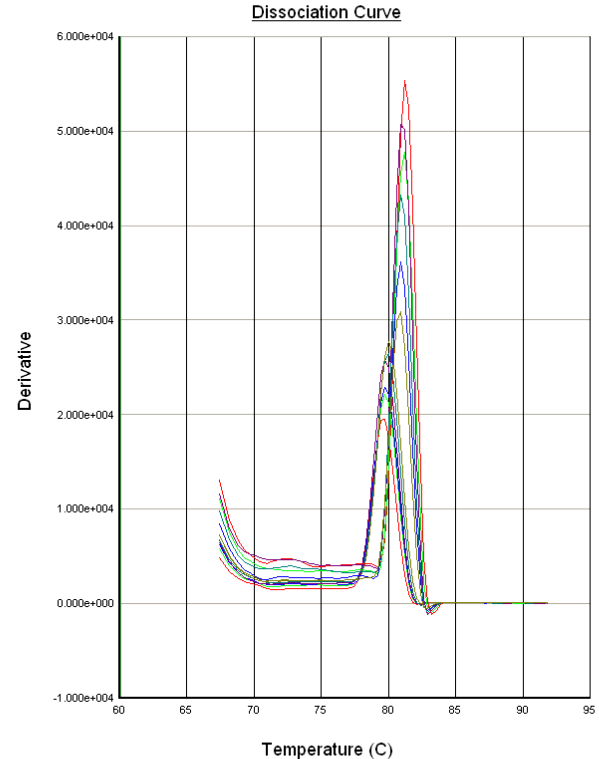 |
| 1. pa010131m |  |
| 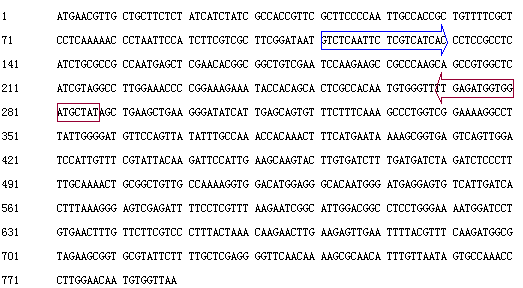 | 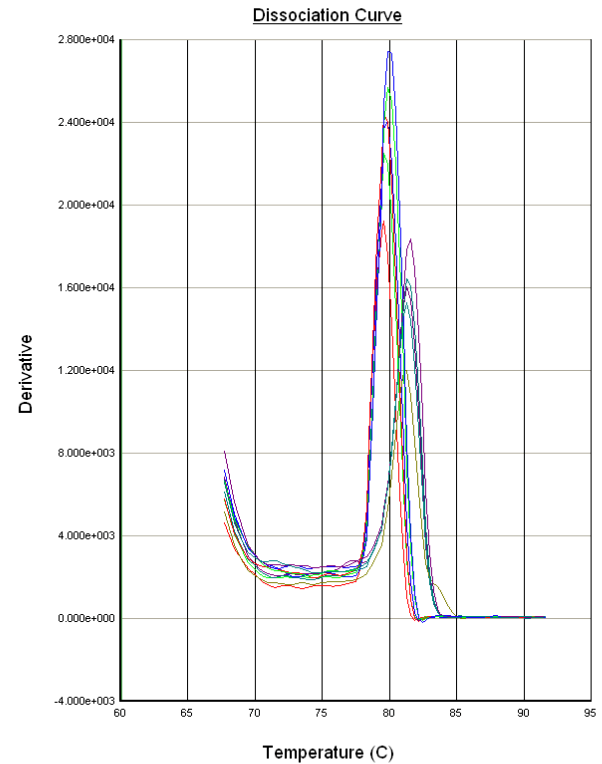 |
| 1. ppa003105m |  |
| 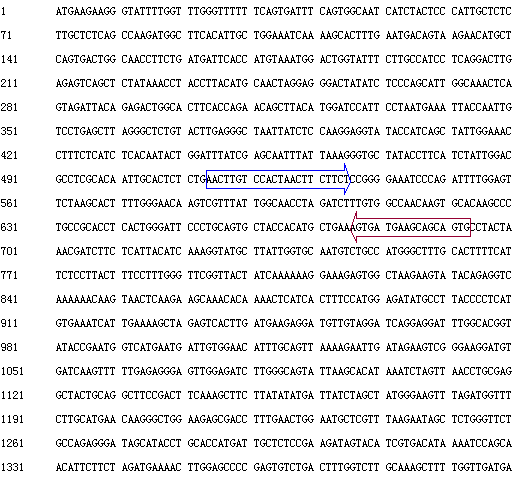  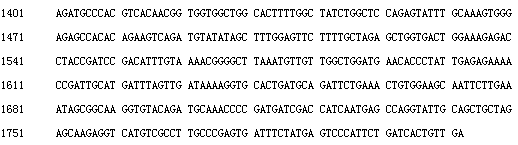 | 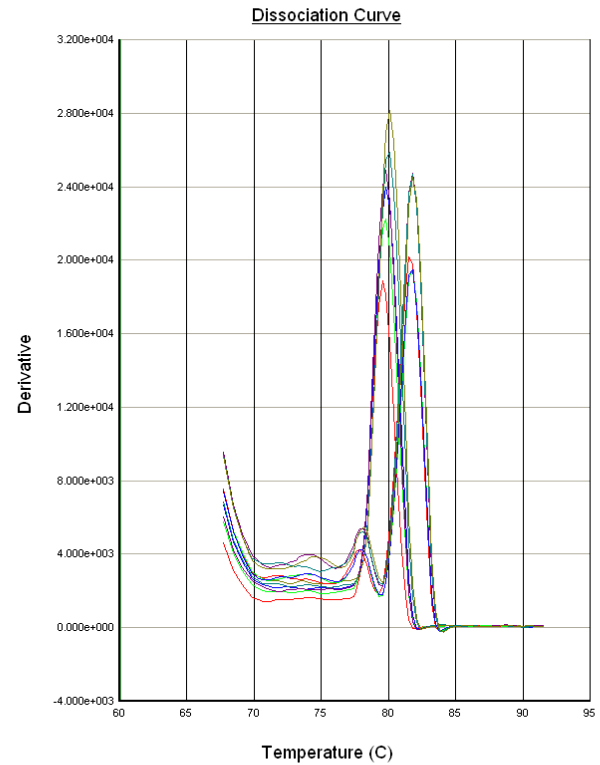 |
| 1. ppa006743m |  |
| 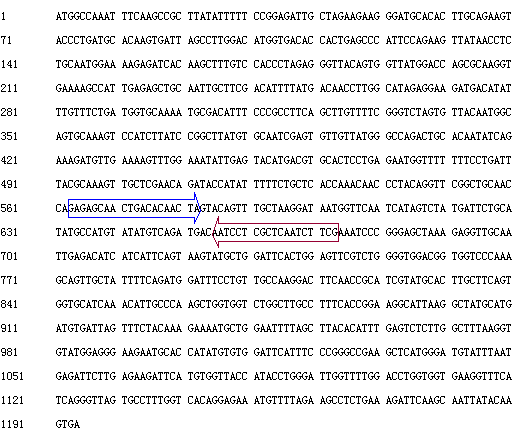 | 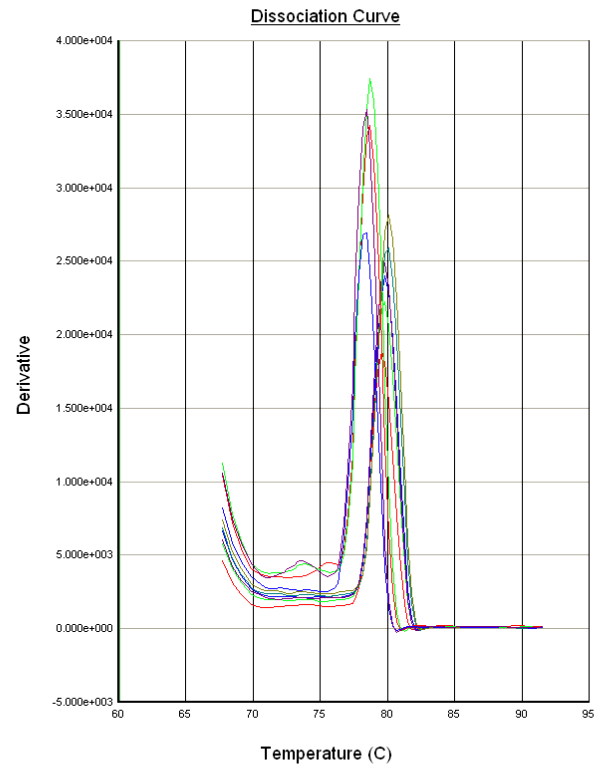 |
| 1. ppa004141m |  |
| 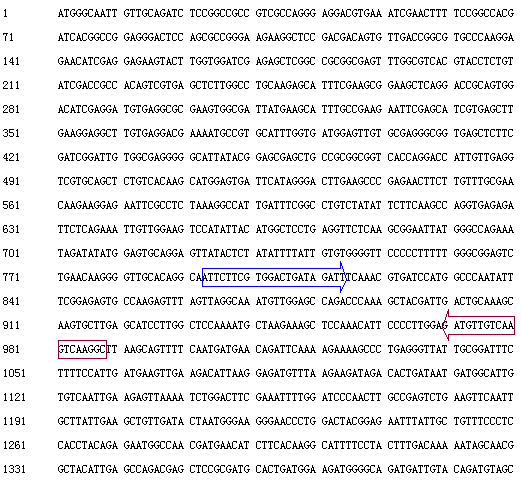  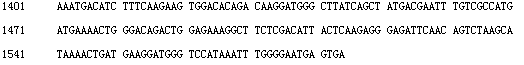 | 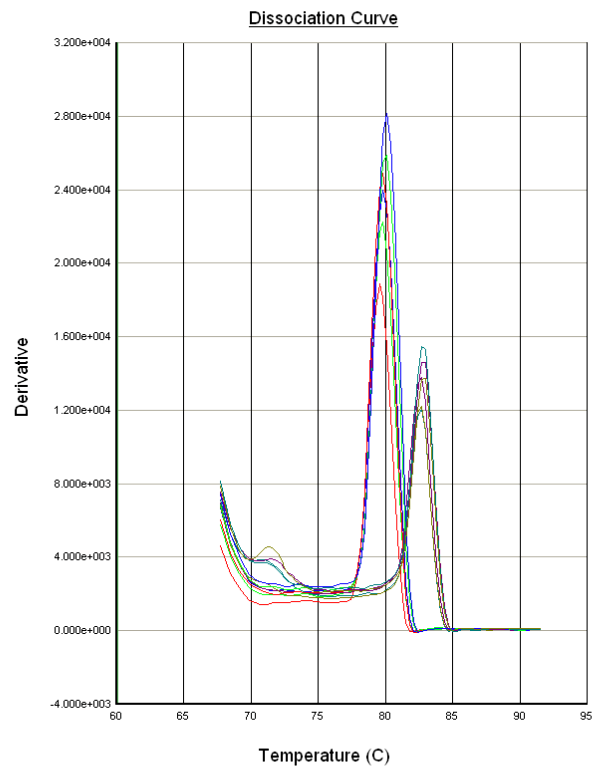 |
| 1. ppa018301m |  |
| 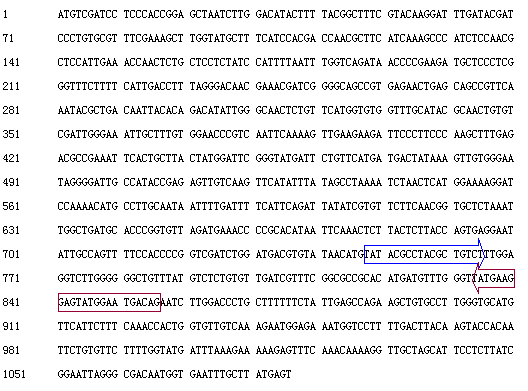 | 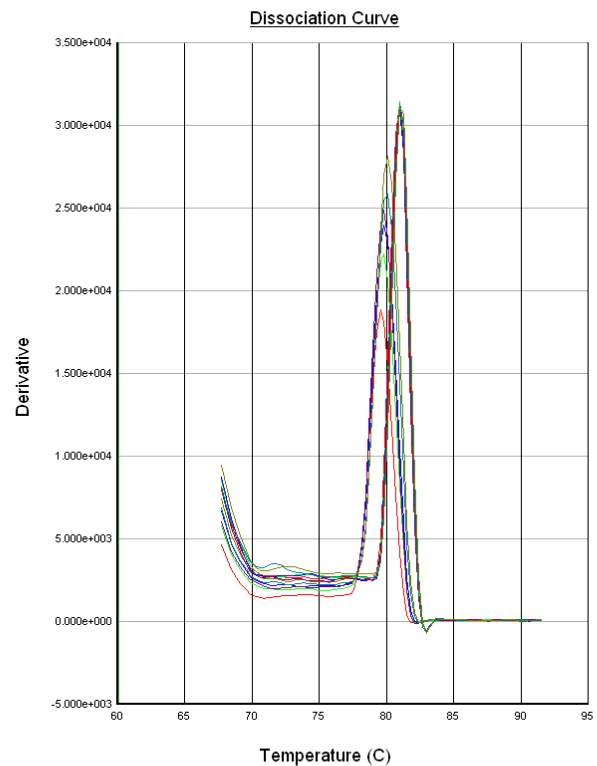 |
| 1. ppa000615m |  |
| 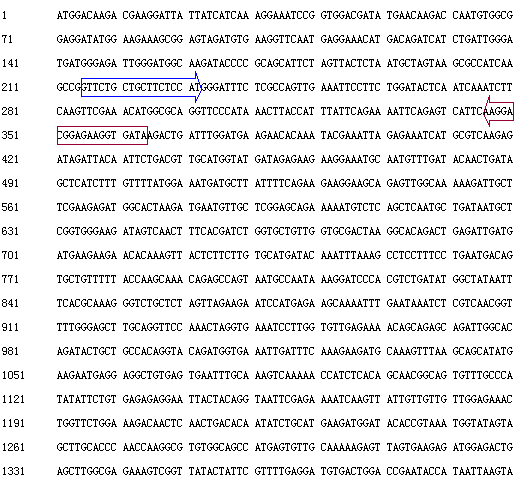  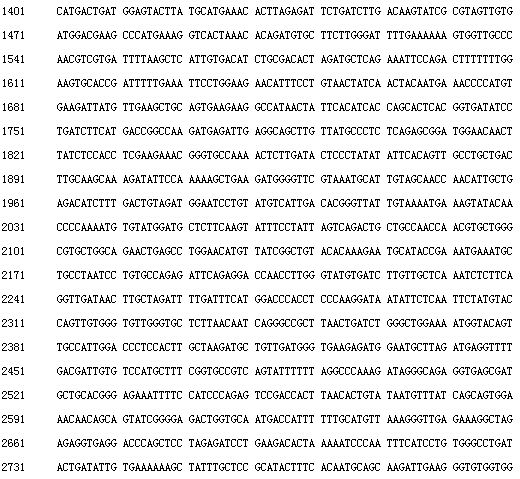  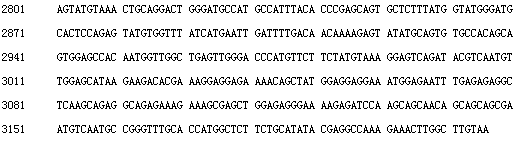 | 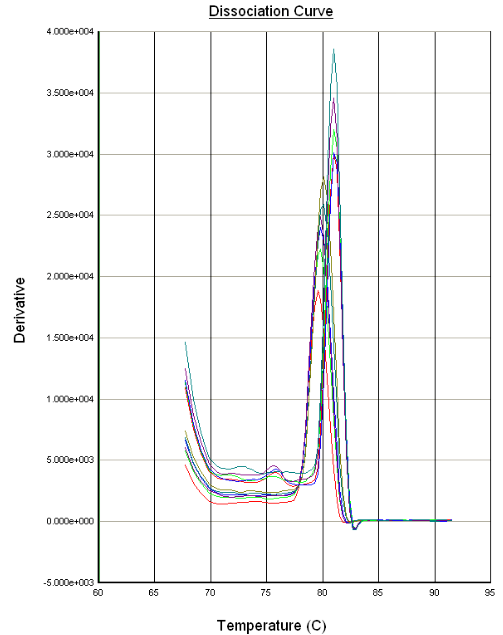 |
| 1. ppa013294m |  |
| 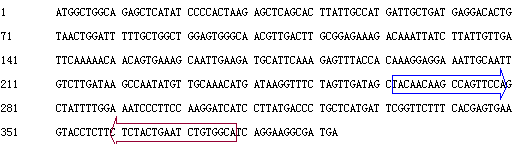 | 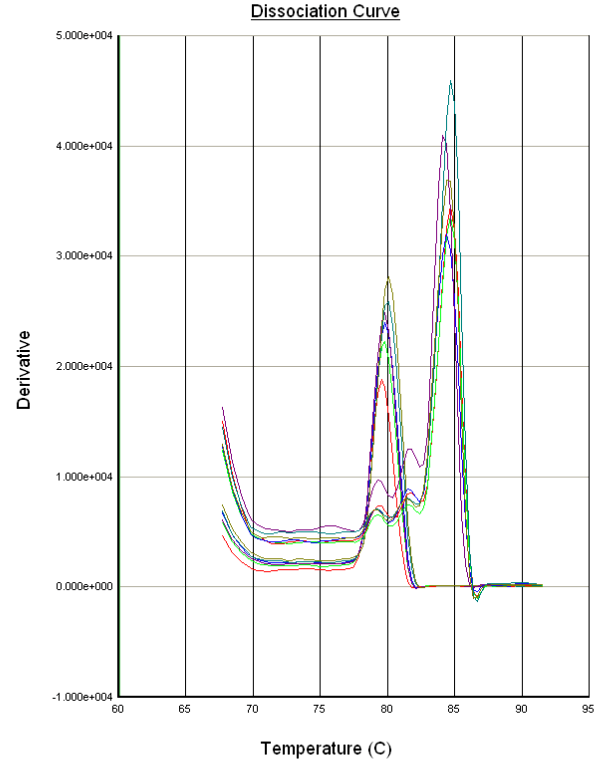 |
| 1. ppa021659m |  |
| 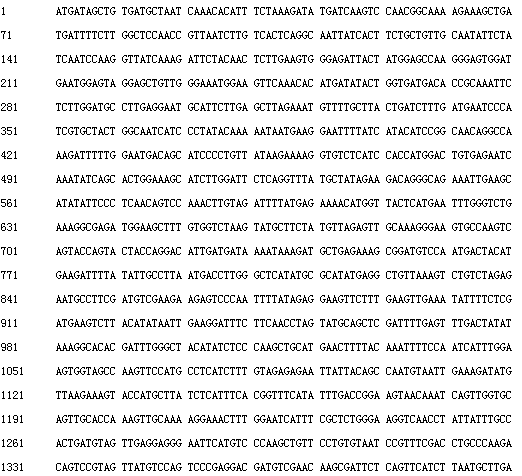  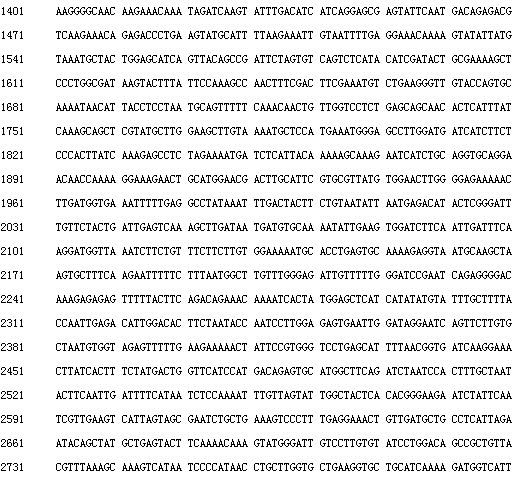  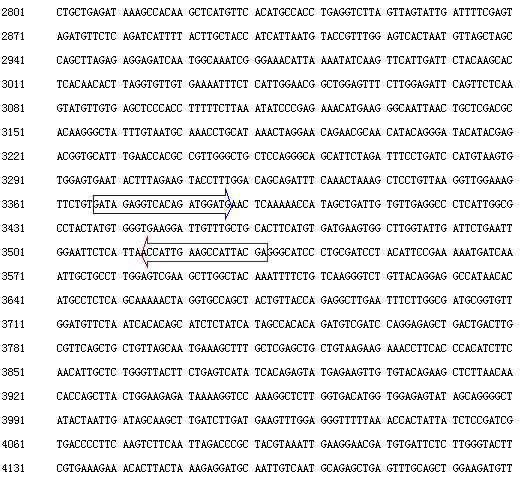  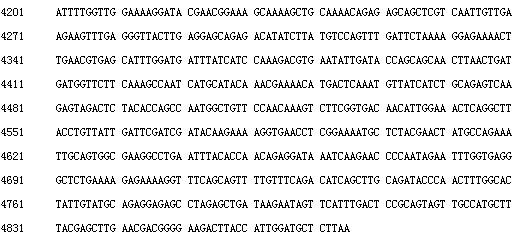 | 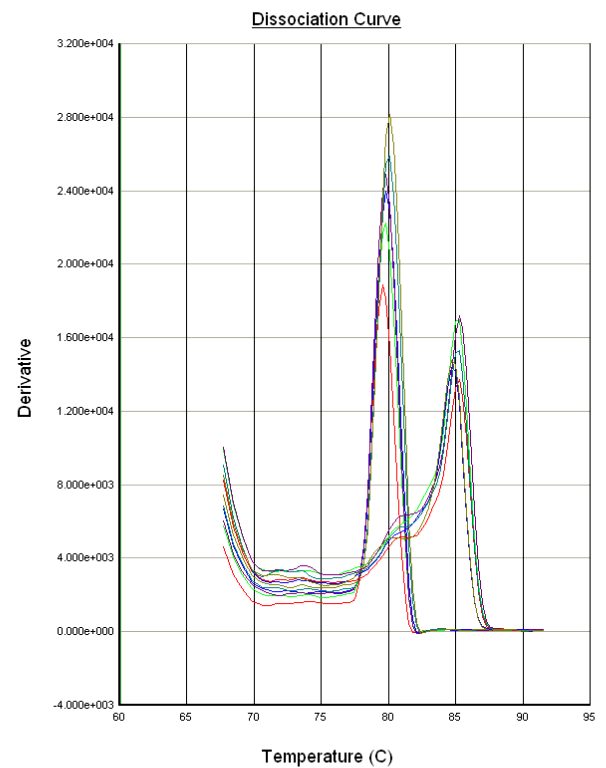 |
